# A rational approach to discovering new persister control agents

**DOI:** 10.1128/aac.01814-24

**Published:** 2025-07-31

**Authors:** Sweta Roy, Zeynep S. Cakmak, Sheila Priscilla Kyeremeh, Shikha Nangia, Juntao Luo, Dacheng Ren

**Affiliations:** 1 Department of Biomedical and Chemical Engineering, Syracuse University 710818, Syracuse, New York, USA; 2 Department of Pharmacology, State University of New York Upstate Medical University 12302, Syracuse, New York, USA; 3 Department of Civil and Environmental Engineering, Syracuse University 710819, Syracuse, New York, USA; 4 Department of Biology, Syracuse University 171413, Syracuse, New York, USA; Shionogi Inc., Florham Park, New Jersey, USA

**Keywords:** persister, killing, drug discovery, chemoinformatic, clustering

## Abstract

Conventional antibiotic drug discovery selects leads based on bacterial growth inhibition. This approach is ineffective against growth-arrested persister cells. When the treatment stops, persister cells revert to normal cells, causing the infection to relapse. To address the challenge of persistent infections, a paradigm shift in antibiotic development is needed to identify new leads that can eradicate dormant cells. Based on our foundational study, we recently proposed a set of principles for developing new persister killing agents. Here, we report the discovery of new leads that are effective against persister cells using a tailored chemoinformatic clustering algorithm based on these principles. We focused on persister penetration using a small compound library that has known antimicrobial activities against normal cells. Experimental testing of eleven compounds identified from clustering led to the discovery of five new compounds that can effectively penetrate and kill persister cells of *Escherichia coli* HM22. The top leads were further tested and also found active against persister cells of *Pseudomonas aeruginosa* and uropathogenic *E. coli* (UPEC), as well as UPEC biofilms and biofilm-associated persister cells. This rather high yield demonstrates the potential of this new rational approach in identifying effective agents against dormant cells, a root cause of persistent infections that is largely missed in conventional screening.

## INTRODUCTION

The antibiotics available to date were developed based on the inhibition of bacterial growth. However, microbes are well known to form phenotypic variants, known as persister cells, that are growth-arrested and thus highly tolerant to most antibiotics (1–3). Upon withdrawal of antibiotics from the surrounding environment, surviving cells can exit from the persister state and resume growth, allowing the infection to persist over time (4, 5). Multiple studies have implicated the presence of persister cells in chronic and recurring infections (4, 5) caused by many bacterial species, such as *Escherichia coli* (6, 7), *Pseudomonas aeruginosa* (8, 9), *Mycobacterium tuberculosis* (10), *Salmonella enterica* subsp*. enterica serovar Typhimurium* (11, 12), and *Staphylococcus aureus* (4, 5). Thus, there is an urgent need for persister control agents and new strategies for antibiotic drug discovery.

Several persister control agents have been discovered in previous screens for killing non-growing cells. For example, Kim et al. (13) screened ~82,000 synthetic molecules using the *Caenorhabditis elegans* methicillin-resistant *S. aureus* (MRSA) infection model and found two synthetic retinoids with potent activities against MRSA persister cells. Other studies screened compounds from drug libraries using persister cells isolated with antibiotics (14–16). These screening methods led to the discovery of several agents that can kill persister cells of *S. aureus* (16) and *Borrelia burgdorferi* (15). However, direct screening of large libraries can be costly and laborious. The lack of a rational approach also hinders the improvement of lead compounds. Thus, we are motivated to develop a new strategy with rational design (based on physicochemical properties of candidate compounds) and guided screening to discover new control agents specifically against persister cells.

The challenge in persister control is mainly attributed to the reduced penetration through the persister cell membrane (4, 17) and the lack of cellular activities that an antibiotic can corrupt upon binding to its target (18, 19). Dormancy of persisters is associated with major changes in cellular activities and membrane functions, e.g., reduced metabolic activities (19–22), decreased membrane potential (17, 23, 24), and changes in membrane fluidity and structure (25, 26). These changes emphasize the need for unique principles to predict penetration through persister membranes, differing from active cells. Unfortunately, the molecular properties needed of a compound that affect its permeability across the bacterial membrane and accumulation in persister cells are poorly understood. This motivated us to identify new leads for persister control by identifying molecular properties that can enhance persister penetration. Recently, we demonstrated that minocycline, rifamycin SV, and eravacycline accumulate more in persisters than normal cells of *E. coli,* leading to effective killing during wake-up (17). Here, we screened a small antimicrobial compound library using eravacycline as the lead, with the goal of identifying new potential persister control agents that can penetrate into these growth-arrested cells and gain more insights toward the discovery of molecular predictors. Our guided screening identified physicochemical properties that are correlated with increased accumulation of compounds in persister cells. Through experimental testing, we discovered five compounds that can effectively kill *E. coli* persisters. In addition, the top leads were found effective in killing uropathogenic *E. coli* (UPEC) persisters and biofilm cells while also showing efficacy against *P. aeruginosa* persister cells. Collectively, these results demonstrate the potential of this rational approach in discovering new control agents against persister cells, addressing an unmet challenge in infection control.

## RESULTS

### A tailored chemoinformatic model to identify leads for persister killing

Recently, we reported a new strategy of persister control by targeting dormancy, the very mechanism that leads to antibiotic tolerance of persister cells (17). Unlike normal cells, persister cells have lower membrane potential (24, 27, 28), and therefore reduced proton motive force and drug efflux (17). Thus, antibiotics that can penetrate bacterial membranes by energy-independent diffusion can accumulate in persister cells, and even more than normal cells if the agent is a substrate of efflux pumps. If the antibiotic binds strongly to its intracellular target, it can kill persister cells upon wake-up when the extracellular antibiotic is removed (before diffusing out or being extruded). This strategy was validated with the finding that minocycline, rifamycin SV, and eravacycline can kill *E. coli* persister cells by 70.8%, 75.0%, and 99.9%, respectively, when treated at 100 µg/mL (17). In comparison, the same treatment with these compounds did not show significant killing of normal cells except for eravacycline (by 63.5%) (17). Based on these findings, we developed several criteria for identifying persister control agents. Specifically, we proposed that persister control agents need to (i) be positively charged under physiological conditions to interact with the negatively charged lipopolysaccharides on bacterial outer membrane, (ii) be able to penetrate persister cells via energy-independent diffusion, (iii) be amphiphilic to have membrane activity for penetration, and (iv) have strong binding to an intracellular target to cause persister killing during wake-up upon removal of extracellular drug molecules (17). Minocycline, rifamycin SV, and eravacycline all met these criteria. In comparison, antibiotics that require active transport to penetrate bacterial membranes would not be effective, e.g., tetracycline, as we reported earlier (17). These criteria are designed based on the phenotypic traits of persister cells. They can be used to select molecular descriptors that can be integrated with computational models to effectively predict persister control agents.

Here, we rationally incorporated this set of criteria in a guided search for new persister control agents using the Asinex SL#013 Gram Negative Antibacterial Library that includes 80 molecules designed based on the iminosugar scaffold (Asinex Corp). This chemical library was chosen as a proof of concept due to its known antimicrobial effects, providing a rational starting point for identifying new persister control agents by focusing on persister penetration. Because persisters are tolerant to conventional antibiotics, they often require compounds with enhanced intracellular accumulation and/or mechanisms that target specific features of dormant cells. Using a library with pre-established antibacterial activity allows us to focus on the properties favorable for persister penetration and thus killing of persisters during wake-up. These compounds have been shown to have growth inhibitory effects on four selected bacterial pathogens, including *Staphylococcus aureus*, *Escherichia coli*, *Pseudomonas aeruginosa,* and *Acinetobacter baumannii* (Asinex Corp.). Iminosugar targets the enzyme *N*-acetylglucosaminidase, which plays a role in peptidoglycan formation of Gram-positive bacteria (29). The mechanism of action of iminosugar or compounds based on the iminosugar scaffold against Gram-negative bacteria is not fully understood yet. However, different derivatives of iminosugar are currently being studied for their effects on processes such as biofilm formation (30, 31), signifying the relevance of these compounds in the antibacterial field. We searched this compound library for molecules with molecular descriptors similar to those of minocycline, rifamycin SV, and eravacycline. This allowed us to narrow down to a subset of compounds that can be tested experimentally for persister penetration and killing.

We first extracted structural and physicochemical parameters from our previously identified persister-killing antibiotics (minocycline, rifamycin SV, and eravacycline) and those from the chemical library using JOELib (32) within the ChemMine platform (33) and the Maestro software (Schrödinger Release 2023-1). These three antibiotics were selected based on prior findings that demonstrated their ability to eradicate *E. coli* persister cells (17). To screen for compounds that align with our criteria mentioned above, we clustered the library using logP (octanol-water partition), halogen content, hydroxyl groups, and globularity. LogP was included given its correlation with compound accumulation in the cytoplasm as shown previously (17, 34). Halogens were considered because some previously discovered persister control agents contain halogens (35, 36). For example, eravacycline, which has a fluorine group and is more effective in persister killing than its precursor minocycline (17). The hydroxyl group was also considered because it contributes to the target binding affinity of many drug molecules (37). Globularity describes the extent to which a molecule adopts a three-dimensional, spherical shape rather than a planar or elongated structure (38). Past studies have reported that low globularity compounds accumulated more in *E. coli* (34, 39). Using these parameters, we applied numeric data clustering followed by the k-means sampling method using the ChemMine platform (33). Since eravacycline demonstrated the strongest persister-killing activity among our reference antibiotics, we focused on the cluster that included eravacycline for further lead selection.

**Fig 1:**
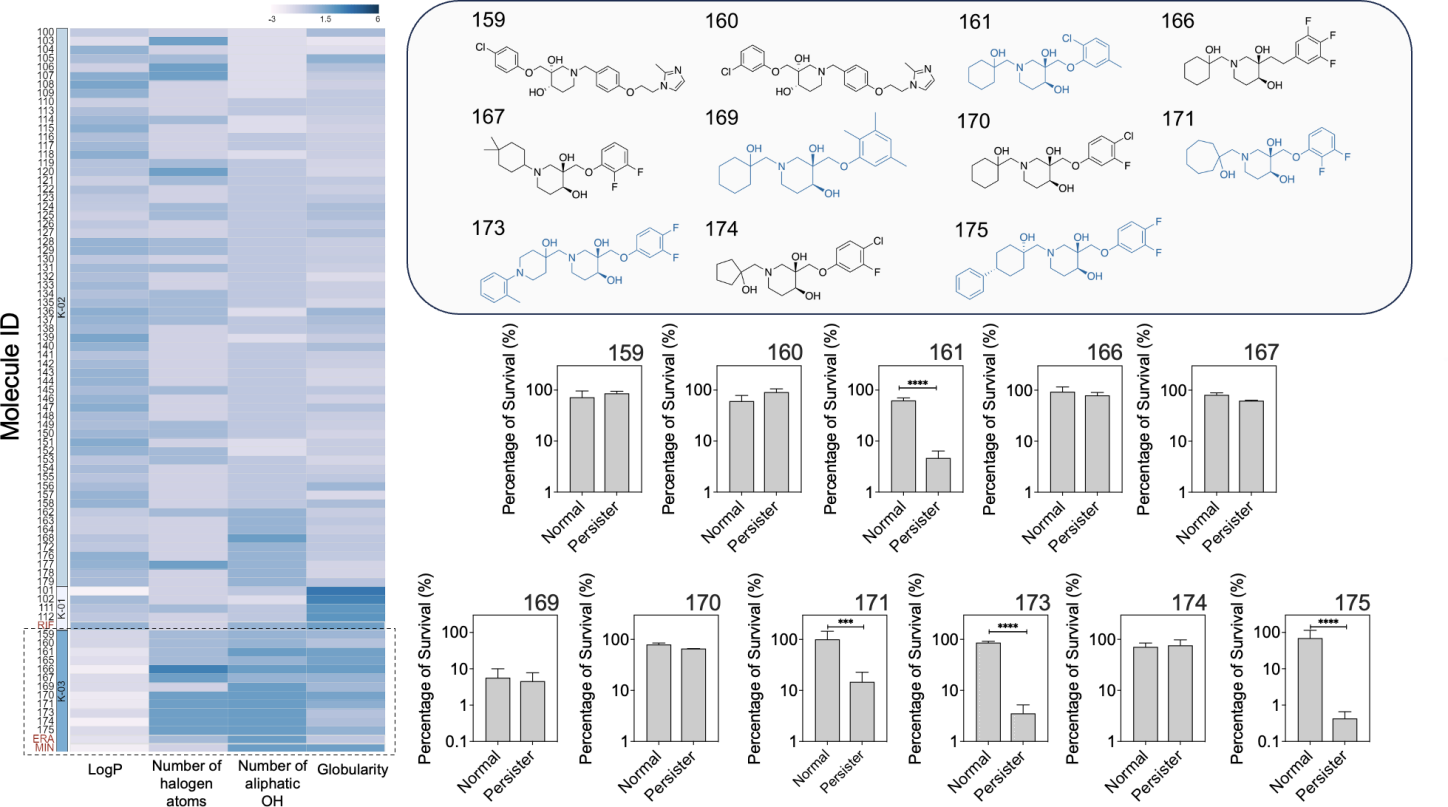
Identification of potential persister control agents using the predictive chemoinformatic model. A screening of 80 compounds was conducted using a cheminformatics-based approach incorporating previously reported persister control agents (minocycline, eravacycline, and rifamycin SV). Compounds were clustered based on structural properties, including logP, halogen atoms, hydroxyl groups, and globularity, leading to the identification of 12 lead compounds that clustered with eravacycline. Among the 11 compounds available for testing, compounds 161, 169, 171, 173, and 175 (highlighted in blue) were found to be effective persister control agents killing 95.5% ± 1.7%, 95.4% ± 1.4%, 85.2% ± 2.7%, 96.4% ± 0.7%, and 98.5% ± 0.5% of the persister population, respectively, when treated at 100 µg/mL in phosphate-buffered saline (PBS) for 1 h. Means ± SE are shown (*n* = 3).

**Fig 2:**
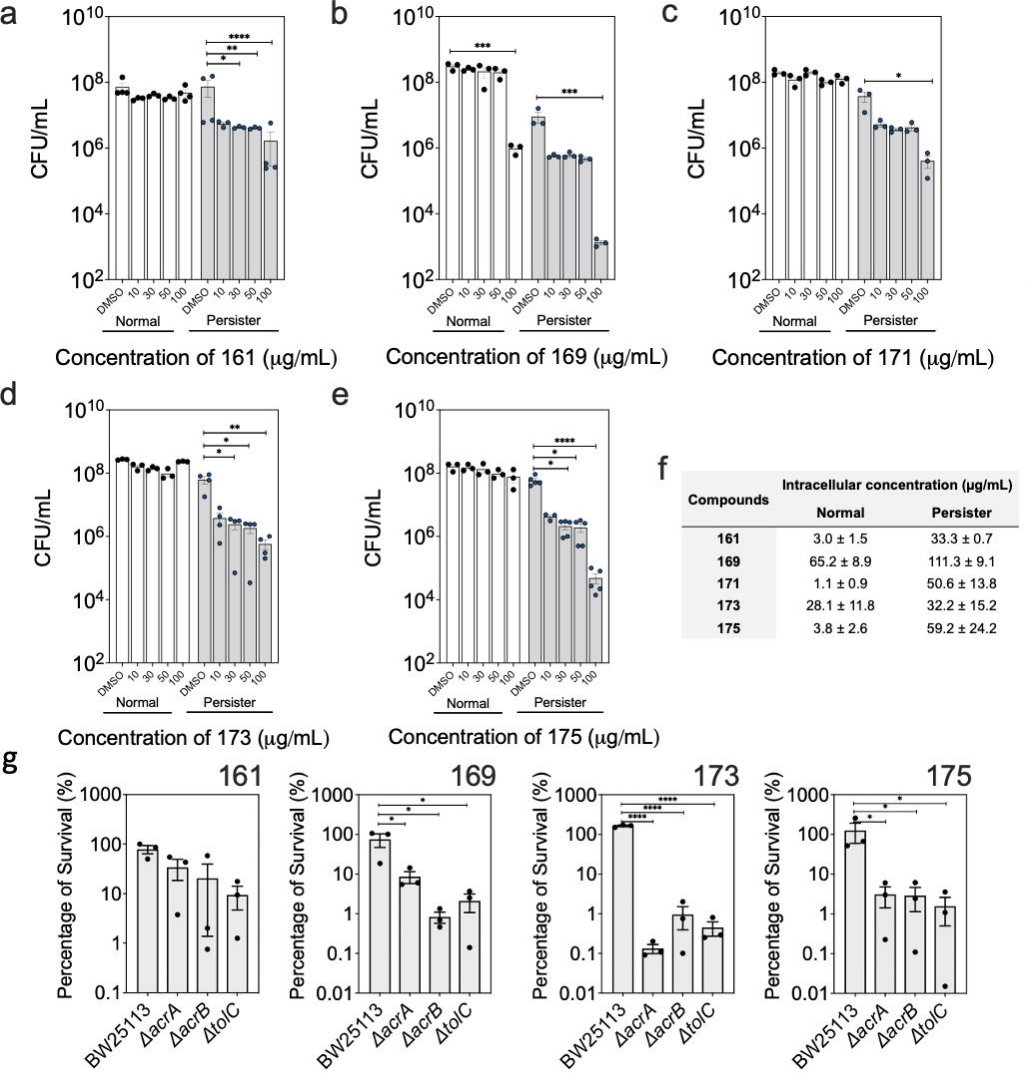
Compounds 161, 169, 171, 173, and 175 are new persister control agents. Effects of compounds 161 (**a**), 169 (**b**), 171 (**c**), 173 (**d**), and 175 (**e**) were tested against normal (white bars) and persister cells (gray bars). The effects were determined based on CFU. Treatment was performed in PBS for 1 h. Means ± SE are shown (*n* = 3). Statistical analysis in **Fig. 2a through e** was performed after transforming into a data as percentage of killing. (**f**) Intracellular concentrations of compounds 161, 169, 171, 173, and 175 in normal and persister cells were quantified after treatment with each compound at 100 µg/mL. (**g**) At the concentration of 50 µg/mL, compounds 161, 169, 173, and 175 were tested for their effects on AcrAB-tolC efflux pump mutants and compared with the wild-type strain BW25113. Means ± SE are shown (*n* = 3).

Twelve compounds clustered with eravacycline and minocycline (Fig. 1). Minocycline also clustered with eravacycline, as expected because its structure is the backbone of eravacycline. Out of the 12 compounds, we tested 11 that were available for purchasing from Asinex (Fig. 2). *E. coli* HM22 was chosen as our model strain because it contains the *hipA7* allele that leads to high-level persistence (40–46), and it was the model strain used in our previous study that identified minocycline, rifamycin SV, and eravacycline as persister killing agents (17). All compounds were tested at a concentration of 100 µg/mL. We observed significant killing of *E. coli* HM22 persister cells by compounds 171, 161, 173, and 175, e.g., by 85.2% ± 2.7%, 95.5% ± 1.7%, 96.4% ± 0.7%, and 98.7% ± 0.5%, respectively (*P* ≤ 0.0001 for all). All four compounds were found to be more effective against persisters than normal cells (Fig. 1). In addition, compound 169 killed 94.4% ± 1.9% of the normal population and 95.4% ± 1.4% of the persister population. As negative controls, we also tested six compounds that did not cluster with minocycline, eravacycline, or rifamycin SV. No significant killing was observed for these six compounds on either normal or persister cells (Fig. S24). These findings help validate the predictive model.

### Further evaluation of the five lead compounds for persister killing

Compounds 161, 169, 171, 173, and 175 showed significant persister killing when treated at 100 µg/mL. To further understand the activities, we tested these five compounds against normal and persister cells of *E. coli* HM22 at different concentrations. Among them, compounds 169 and 171 only exhibited persister killing at 100 µg/mL, indicating a threshold concentration is required. In comparison, dose-dependent killing of persisters was observed for compounds 161, 173, and 175 (Fig. 2a, d and e). For example, compound 173 showed 65.9% ± 5.0% (*P* = 0.02), 74.2% ± 3.6% (*P* = 0.01), and 96.4% ± 0.7% (*P* = 0.003) killing of persister cells at concentrations of 30, 50, and 100 µg/mL, respectively. Based on these results, 100 µg/mL appeared to be a general threshold for substantial killing. Compared to the cidal effects on persister cells, no significant killing of normal cells was observed under the same conditions, except for 94.4% ± 1.9% killing (*P* < 0.0001) by compound 169, when treated at 100 µg/mL in phosphate-buffered saline (PBS).

To corroborate the persister killing results, we evaluated the penetration of compounds 161, 169, 171, 173, and 175, which exhibited more than ~85% killing of persister cells (Fig. 2a through e). Consistent with the viability results, compounds 161, 169, 171, and 175 all accumulated more in persister cells than normal cells (Fig. 2f). It is interesting that although the accumulation of compound 173 was similar between normal (28.1 ± 11.8 µg/mL) and persister (32.2 ± 15.2 µg/mL) cells, more killing of persisters (96.4%) than normal cells (13.5%) was observed, indicating that persister cells may have a higher abundance of the target or other targets for compound 173. The difference could also be due to different mechanisms of action, non-specific growth inhibition, or slow off-rate.

### Mutation of the AcrAB-TolC efflux pump increased susceptibility to lead compounds

To better understand how persister killing occurred, we tested the minimum inhibitory concentration (MIC) of compounds 161, 169, 171, 173, and 175, which exhibited more than ~85% killing of persister cells. The results show that all five compounds have an MIC >128 µg/mL in Lysogeny broth (LB) medium. We speculate that this is due to efflux activities (compared to strong killing of efflux mutants in Fig. 2g), and thus different effects on persister vs normal cells.

In our previous study (17), reduced drug efflux was found as a contributing factor to persister killing by eravacycline, minocycline, and rifamycin SV. Because compounds 161, 169, 173, and 175 accumulated significantly in persister cells, we speculate that the dormancy-related decrease in efflux may also play a role. To test this, we evaluated whether these compounds could kill normal cells of multidrug AcrAB-TolC efflux pump mutants (17, 47). Indeed, we observed increased killing of all three efflux mutants compared to the wild-type strain by compounds 161, 169, 173, and 175. When treated at a concentration of 50 µg/mL, none of the four compounds had significant killing of normal cells of the wild-type strain. However, compound 169 showed killing of 91.4% ± 2.3% (*P* = 0.02), 99.2% ± 0.2% (*P* = 0.02), and 97.9% ± 0.8% (*P* = 0.02) of *ΔacrA*, *ΔacrB*, and *ΔtolC* mutants*,* respectively (Fig. 2g). Similar killing activities by compounds 173 and 175 were observed for the same three mutants (Fig. 2g). Compound 161 showed more than 60% killing of *ΔacrA*, *ΔacrB,* and 90.2% ± 3.8% killing of *ΔtolC* mutants (Fig. 2g). These findings indicate that reduced efflux activities of persister cells may contribute to the killing by these compounds. We speculate that the reduced efflux allowed the compounds to penetrate into persister cells and accumulate, leading to killing during wake-up. These results also indicate that normal cells may extrude these compounds, which explains why these compounds are less effective against normal cells.

While these effective compounds showed increased accumulation in persister cells, the mechanism of killing is unknown. These compounds are based on iminosugar scaffolds, which target N-acetylglucosaminidase involved in cell wall recycling of Gram-positive bacteria. To determine whether these chemical compounds target N-acetylglucosaminidase of *E. coli*, we further tested the leads using a *nagZ* mutant strain. The *nagZ* gene encodes a β-N-acetylglucosaminidase involved in peptidoglycan recycling (48). We thus compared the wild-type, BW25113, and its *nagZ* mutant from the Keio collection (49). The mutation did not show a clear effect on persister killing compared to the wild-type strain (Fig. S27 and S28), suggesting the presence of other targets in *E. coli*.

### Effects of amphiphilic moment and globularity on persister killing

**Fig 3:**
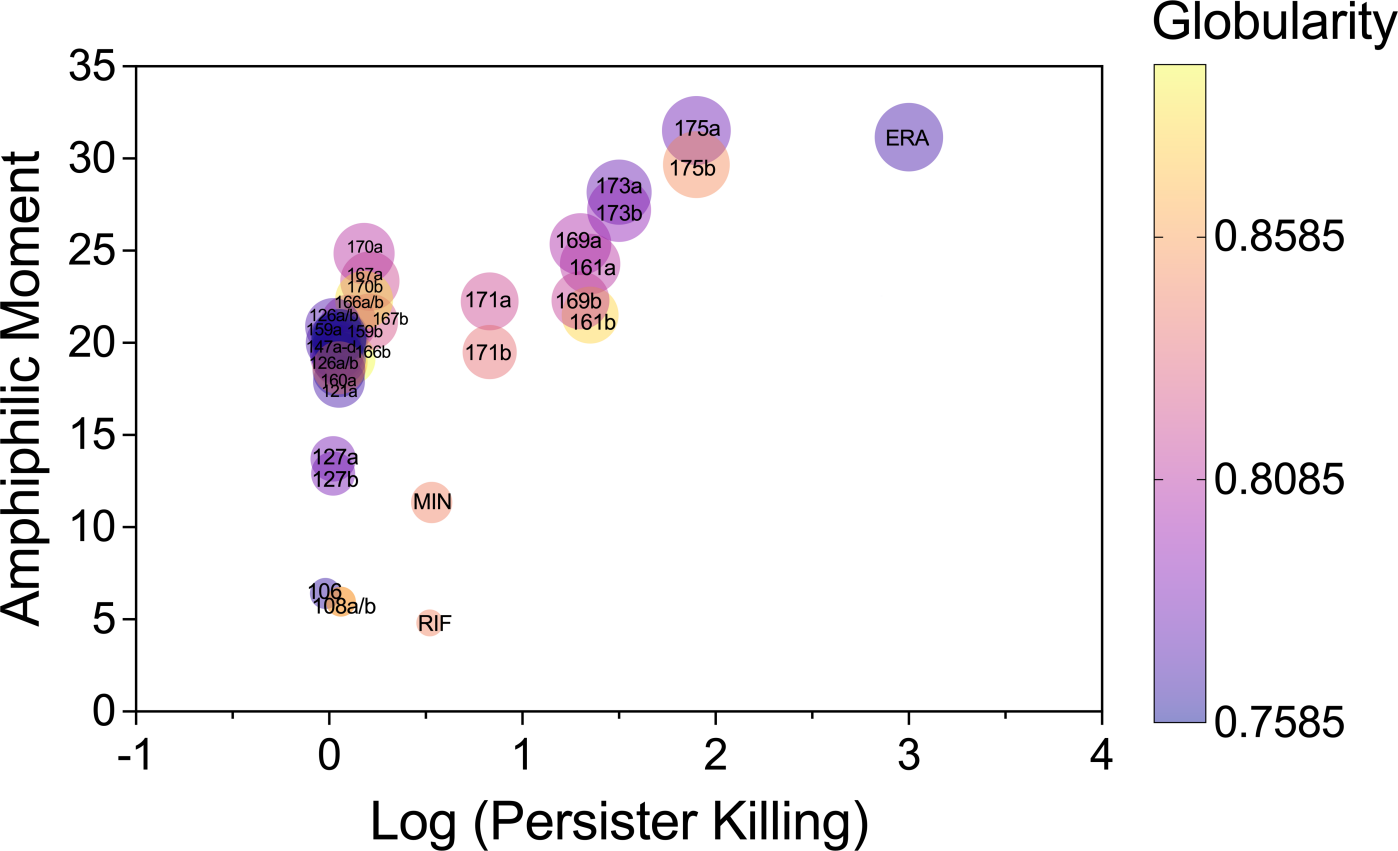
Relationship between amphiphilic moment, globularity, and persister killing. Persister killing data for each of the 11 tested compounds and the six negative controls at 100 µg/mL (as reported in Fig. 2; Fig. S24) are plotted against their calculated amphiphilic moment. The amphiphilic moment was determined based on the 3D structure of each compound, including potential structural variants (denoted as a, b, c, or d). The color gradient represents globularity values, providing insight into how molecular shape may contribute to persister killing.

To further understand what features the five compounds may have in common that led to persister killing compared to the other compounds within this cluster, the amphiphilic moment was calculated and compared with globularity and persister killing. Amphiphilic moment measures the distance between the hydrophobic and hydrophilic centers of the compound (34) and can contribute to how a compound interacts with and penetrates through cell membranes. All four compounds that had more than 85% killing of the persister population have high amphiphilic moment values. The compounds that had more than 1.5 logs of persister killing (173 and 175) have higher amphiphilic moments (>25) than other compounds and close to that of eravacycline. In addition, the globularity of the same compounds is in the mid-range, with a value of 0.8 determined using the Schrödinger software (Fig. 3). Together, these findings indicate that a high amphiphilic moment value may be desirable for effective persister control agents. Further testing of a larger library will help validate and refine these criteria.

### Compounds 173 and 175 are effective against persisters of other Gram-negative bacteria

**Fig 4:**
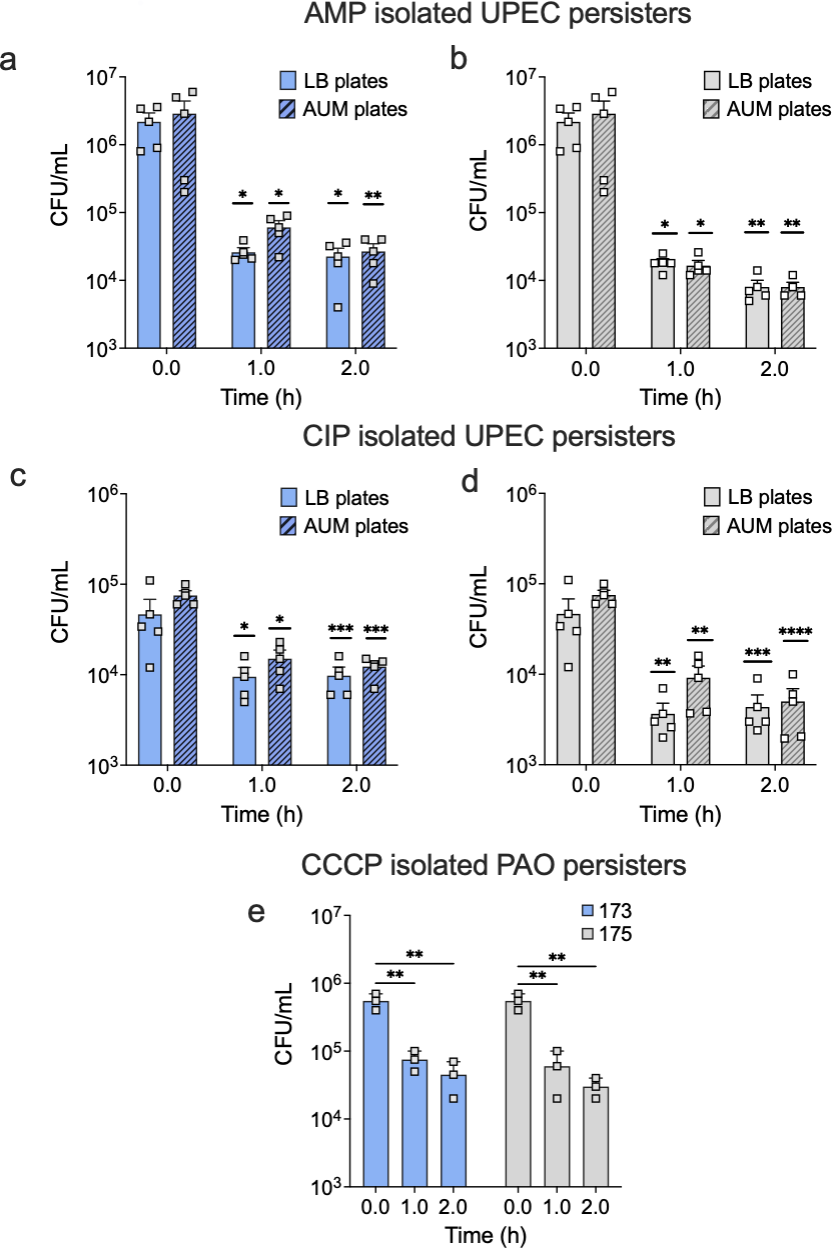
Compounds 173 and 175 have significant activities against UPEC and PAO1 persister cells. UPEC persister cells were isolated using 100 µg/mL ampicillin (AMP) (**a, b**) or 1 µg/mL ciprofloxacin (CIP) (**c, d**) and then treated with 100 µg/mL of compound 173 (**a, c**) or 175 (**b, d**) for 1 or 2 h in PBS. The samples were washed and plated on either LB or AUM plates to determine CFU (*n* = 4). *P. aeruginosa*, PAO1 persister cells were isolated after 3.5 h incubation with cyanide m-chlorophenylhydrazone (CCCP) at a concentration of 200 µg/mL. After persister isolation, cells were washed and then treated with 100 µg/mL of compound 173 (**e**) and 175 (**e**) for 1 and 2 h in PBS. Viability was determined after treatment based on CFU (*n* = 3). Time 0 refers to the untreated control.

Given the promising activities of the compounds on a lab strain, *E. coli* HM22, we sought to determine if the top leads are effective against persister cells of the pathogen UPEC. Compounds 173 and 175 were selected as the top leads because these two compounds exhibited the highest persister-killing activities, e.g., more than 95% killing of *E. coli* HM22 persisters. Additionally, both compounds demonstrated dose-dependent killing, further supporting their potency. UPEC is a major causative agent of urinary tract infection (UTI) (7), which has a high recurrence rate of 25% (50). UPEC persisters were isolated using 100 µg/mL of ampicillin (AMP) or 1 µg/mL of ciprofloxacin (CIP) (Fig. S23). Both compounds showed significant killing of UPEC persisters isolated by AMP or CIP. For example, at the concentration of 100 µg/mL (2 h in PBS), compound 173 caused 99.0% killing of AMP persisters (2.3 ± 0.6 × 10^4^ vs 2.2 ± 0.7 × 10^6^, *P* = 0.04) and 80.0% killing of CIP persisters (9.7 ± 2.0 × 10^3^ vs 4.7 ± 1.9 × 10^4^, *P* = 0.04), compared to the control (Fig. 4a through d). A similar trend was observed for compound 175 when treated at 100 µg/mL in PBS for 2 h. It caused 99.6% killing of AMP persisters (8.0 ± 1.3 × 10^3^ vs control, *P* = 0.04) and 90.7% killing of CIP persisters (4.4 ± 1.8 × 10^3^ vs control, *P* = 0.007) (Fig. 4a through d). We speculate that the slightly higher tolerance of CIP persisters than AMP persisters may be due to the longer lag time for CIP isolated persisters to wake-up (Fig. S26) and thus more time for intracellular drug to diffuse out before cell killing can happen. The findings described above demonstrated that the leads were able to accumulate inside persister cells. To determine if the wake-up medium affects persister killing, we plated treated persister cells on agar plates of different media. Artificial urine medium (AUM) was used for UPEC experiments because it mimics the *in vivo* environment of urinary tract infections. Our data indicate that at the concentration of 100 µg/mL, treatment with compounds 173 and 175 caused 99.1% ± 1.6% (*P* = 0.009) and 99.7% ± 0.8% (*P* = 0.009) killing of AMP persister cells and 83.7% ± 8.6% (*P* = 0.0003) and 93.3% ± 2.2% (*P* = 0.0001) killing of CIP persister cells, respectively (Fig. 4a through d). These results highlight how the environment affects the potency of the compounds in persister control.

To further understand if persister killing occurred during wake-up, we followed UPEC persister cell resuscitation after treatment (compounds 173 and 175 tested). To explore how the resuscitation dynamics vary, we monitored regrowth in both LB and AUM, including AUM supplemented with metabolites such as glucose (20) and/or L-alanine (51), which were previously reported to accelerate persister wake-up. The results show an increase in the lag phase during resuscitation of treated persister cells compared to untreated controls (Fig. S29). To further validate our resuscitation results, we performed a flow cytometry-based analysis using SYTO9/PI staining to monitor regrowth dynamics in treated and untreated persister cells of *E. coli* HM22 over time. Consistently, we found the SYTO9/PI ratio of persister cells treated with compounds (161, 169, and 175 tested) to be lower than untreated control, especially during the time window of initial regrowth of untreated cells (Fig. S30). The similar SYTO9/PI ratios between treated and untreated cells at time 0 (right after treatment) indicate that killing did not happen during the 1 h treatment time, but rather during regrowth. Collectively, these results support our model that persister killing can be achieved during wake-up with internalized antimicrobials.

To further characterize compounds 173 and 175 for persister killing, we tested the potency of these compounds against persister cells of *P. aeruginosa. P. aerugi*nosa is a major bacterial pathogen due to its remarkable capabilities to cause chronic and life-threatening infections in humans, such as wound infections, catheter-associated infections, and more (8, 9, 52–54). Persister cells were isolated from *P. aerugi*nosa PAO1 cultures using 200 µg/mL cyanide m-chlorophenylhydrazone (CCCP), as previously described (55, 56). When treated at 100 µg/mL, compounds 173 and 175 killed 91.8% ± 3.2% (*P* = 0.007) and 94.5% ± 1.3% (*P* = 0.006) persisters in 2 h, respectively (Fig. 4e). This indicates that the top lead compounds are also effective against other Gram-negative bacteria such as *P. aeruginosa*.

### Compounds 173 and 175 are effective against UPEC biofilms and associated persister cells

**Fig 5:**
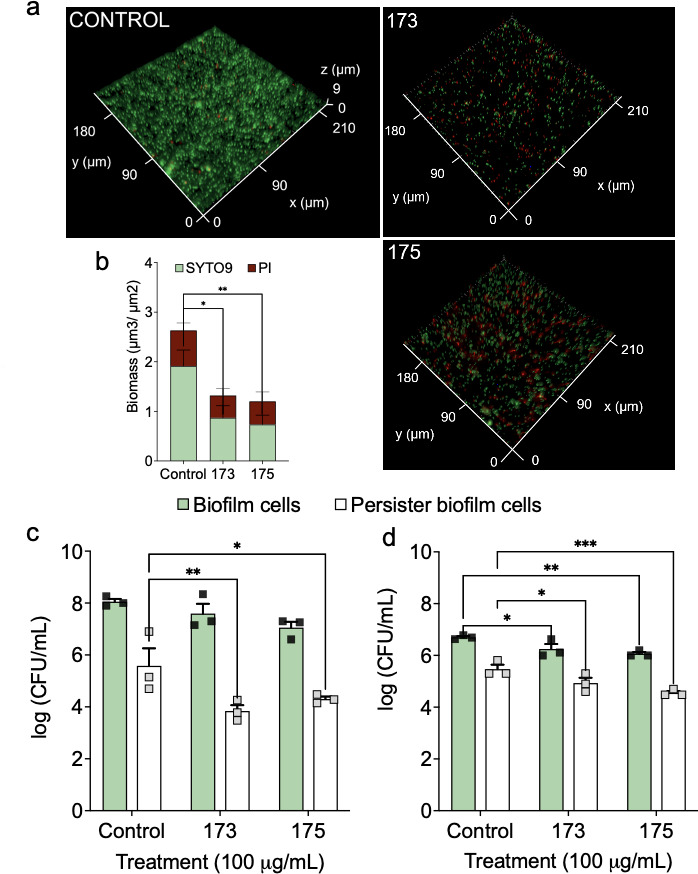
Compounds 173 and 175 killed biofilm cells and biofilm-associated persister cells. (**a**) Fluorescent images of control and treated biofilms. The 48 h biofilms were stained with SYTO9 and PI and imaged after treatment with compound 173 or 175. (**b**) Biomass was quantified using COMSTAT; *n* = 3. (**c and d**) Viability of the treated biofilms (green bars) was determined based on CFU after plating on either LB agar plates (**c**) or on AUM agar plates (**d**) (*n* = 3). Persister cells of biofilm were quantified (white bars) by treating the detached biofilm cells with 100 µg/mL ampicillin for 3.5 h and plating them on either LB agar plates (**c**) or on AUM agar plates (**d**); *n* = 3.

UPEC biofilms formed on host epithelial cells and urinary catheters (57) are difficult to eradicate. Since compounds 173 and 175 are effective against UPEC persister cells, we further tested their effects on 48 h UPEC biofilms cultured in AUM. Tested at 100 µg/mL for 1 h, both compounds reduced biomass significantly, e.g., 54.6% ± 27.4% and 61.6% ± 21.2% by compounds 173 and 175, respectively (Fig. 5a and b). Killing of biofilm cells was corroborated by propidium iodide (PI) staining (Fig. 5a and b). This was also validated with CFU (Fig. 5c and d). When plated on LB plates, compounds 173 and 175 killed 46.8% ± 28.2% and 87.0% ± 5.0% of the biofilm cells, respectively, compared to the control (Fig. 5c). When plated on AUM plates, compounds 173 and 175 showed 59.7% ± 12.8% and 76.5% ± 1.5% killing, respectively (Fig. 5d). Interestingly, the compounds were more effective against biofilm-associated persister cells compared to the overall biofilm cells, consistent with their stronger effects against persisters than normal cells in planktonic samples discussed above. Specifically, compound 173 killed 99.7% ± 3.0% (*P* = 0.004) and 69.5% ± 4.7% (*P* = 0.02) biofilm-associated persisters when treated cells were plated on LB and AUM plates, respectively. Similar killing by compound 175 was observed, e.g., 99.2% ± 9.2% (*P* = 0.02) and 89.5% ± 4.8% (*P* = 0.0006) for the two plating conditions, respectively (Fig. 5c and d). Overall, these results demonstrate significant activities of compounds 173 and 175 against UPEC biofilms and associated persister cells.

## DISCUSSION

Increasing evidence emphasizes the urgent need for more effective agents to control persistent infections. Persister cells are growth-arrested; thus, most antibiotics that were selected to target active cells fail to eradicate persisters. In this study, we identified new leads for persister control based on a set of criteria we developed recently (17). The results demonstrate an efficient approach to identifying persister control agents that are able to penetrate into the cells using a guided screening that incorporates phenotypic traits with molecular descriptors. Our workflow includes combining the set of criteria with the reported persister control agents from our previous study to predict possible leads in persister penetration. This strategy was validated using the Asinex chemical library with known antimicrobial activities, and compounds 161, 169, 171, 173, and 175 were identified as effective leads for persister killing. The high hit rate (5 out of 11) demonstrates the capability of this predictive chemoinformatic model for searching persister control agents. To further evaluate, the top two leads (173 and 175) that exhibited more than 95% killing were tested against UPEC persister cells, including biofilm-associated persisters. Significant activities were observed for both compounds. In addition to rich (LB) medium, the activities were also observed in AUM, indicating potential applications in controlling UTI and catheter-associated infections. Furthermore, the top leads also showed significant activities against *P. aeruginosa* persister cells, making the two compounds promising persister control agents.

The parameters used to identify the new compounds in this study are based on the low membrane potential of dormant cells (17, 27, 28, 58–66). Since most efflux pumps rely on proton motive force to function, which is governed by the change in membrane potential and pH (27), we expect that dormant cells have reduced drug efflux (17). Meanwhile, dormant cells allow penetration of antibiotics that can enter the cells by diffusion through lipid membranes. This would lead to the accumulation of such antimicrobials in persisters, even if they are substrates of efflux pumps. An earlier study reported enhanced efflux in *E. coli* persister cells (67); however, the conclusion was made on a heterogeneous (68, 69), rather than a specific population of dormant cells. Using our new strategy that we previously reported (17, 70), we identified parameters to redefine the search for new persister control agents by using the Simplified Molecular Input Line Entry System (SMILES) profile of the chemical compounds to predict related physicochemical characteristics. This differs from the traditional screening, which relies heavily on growth inhibition and thus is ineffective against dormant cells. Our study also highlights what molecular descriptors need to be considered to design new antibiotics for persister penetration. Past studies have identified that most active antibiotics against *E. coli* can be classified by molecular descriptors that include topology, atom counts, or bond counts (39). Amphiphilicity is also an important property to consider for antibiotic accumulation (34, 39). While amphiphilic moment alone does not universally correlate with persister penetration, our analysis indicates that compounds with log-transformed persister killing greater than 1.5 (173 and 715) both exhibit high amphiphilic moments (>25) like eravacycline, suggesting that there may be a threshold of amphiphilicity for strong persister penetration. Compounds 161, 169, 173, and 175 (this study) and the antibiotic eravacycline (17) exhibited increased accumulation in persister cells compared to minocycline and rifamycin SV (17). While these compounds displayed higher amphiphilic moments compared to most of the other tested compounds (Fig. 2), the relationship between amphiphilicity and intracellular accumulation is not directly correlated. For example, compound 169 showed the highest accumulation but had a lower amphiphilic moment than 173 and 175, suggesting that other factors, such as efflux susceptibility or intracellular binding, may also affect accumulation. Likewise, compounds 171 and 175 displayed similar accumulation levels despite their differences in amphiphilic moment. These findings indicate that amphiphilicity likely contributes to drug accumulation but is not the sole determinant of intracellular concentration. Future studies should further investigate how multiple physicochemical properties, including efflux evasion and target binding, collectively impact persister cell penetration and intracellular accumulation.

Another important molecular descriptor to consider for potential persister control agents is globularity (34, 39). It is important to note that the globularity calculation varies depending on the methodology used. In this study, globularity was determined using the Schrödinger software, which calculates molecular globularity and describes the molecular volume and 3D shape of the compound based on solvent-accessible surface area and van der Waals radii (71) (Maestro User Manual, Schrödinger Release 2023-1, LLC, New York, NY 2023). Using this method, the active leads had globularity values in the range of 0.8–0.82, indicating a degree of spherical shape. The shape of a molecule affects its interaction with other components in biological systems and is often used to assess molecular transport across barriers such as skin. Alternatively, another approach to globularity, the eNTRy rule, defines globularity based on the molecular shape, calculated as the inverse condition number of the covariance matrix of atomic coordinates (34). Using the eNTRy rule calculation, our active leads exhibited low globularity (0.06–0.13), aligning with prior studies that suggest compounds with lower eNTRy globularity tend to accumulate more efficiently in *E. coli* (34). This distinction highlights the importance of considering both molecular volume and molecular shape when evaluating compound penetration into persister cells.

With the presented results, we demonstrate the feasibility of using a guided rational search for new persister control agents that are able to penetrate and accumulate in these dormant cells. This approach provides a framework for prioritizing a small set of high-potential candidates, enabling future studies to efficiently test compounds that may target and eradicate persister cells, rather than randomly screening entire chemical libraries in a costly and time-intensive manner. This approach can be modified for more comprehensive screens, e.g., applying the same flowthrough on a much larger and diverse set of chemical compound libraries where all three positive candidates and other known persister control agents can be included. Using the SMILES profiles of the chemical compounds, physicochemical characteristics can be predicted based on our algorithmic workflow, which incorporates cheminformatic models to evaluate properties such as logP, globularity, and halogen groups. In addition, it would be interesting to see if the parameters identified in this study, such as globularity or amphiphilic moment, correlate with already identified persister control agents. During the preparation of this manuscript, Zheng et al. (72) reported a new study that screened ~5 million compounds from a Repurposing Drug depository by applying deep learning screening with lethality against metabolic dormant cells, an approach that minimizes laborious cost. The outcome of their study led to the discovery of semapimod as a potent agent against dormant cells, along with other agents such as eravacycline, bekanamycin, LTX-315, and BAI1 (72). It is intriguing to see eravacycline identified in a totally different search. Meanwhile, the low hit rate indicates that commercially available compounds have very limited persister control capabilities. The results from the present study demonstrate the potential of guided screening based on the principles we recently proposed for persister control (17). Using this rational approach, it is possible to reduce the library size for screening and enable guided design of derivatives based on selected core structures.

This study focuses on persister penetration by leveraging a compound library that has known antibacterial activities. The compounds in the Asinex library are based on iminosugar scaffolds. It has been reported that iminosugar targets N-acetylglucosaminidase, which plays a role in cell wall recycling of Gram-positive bacteria. The effects on Gram-negative bacteria are not well understood. However, the lack of effects of the *nagZ* mutation (Fig. S27 and S28) suggests the presence of other targets in *E. coli*.

To move this approach beyond just predicting penetration and toward actual persister killing, we also need to consider factors such as target residence time, target turnover rate, and the mechanism of killing. Identifying key predictors for target binding and optimizing the structure of lead compounds to enhance antibacterial efficacy is a part of our future work. The binding affinity can be evaluated through simulations, which will enable further improvement to refine the leads based on these predictive insights. Additionally, expanding this approach to a larger, structurally diverse library will allow for broader validation of its effectiveness. Antibacterial activities are also required for persister killing during wake-up. This can be rapidly screened using an efflux mutant. Persister treatments were tested in PBS for 1 h in this study. Further studies should include longer treatment times under more physiologically relevant conditions, which will help understand if there are adaptive responses of bacteria through mutations.

Developing new antimicrobials that do not require metabolic activities of the targeted cells to function is desirable because they are effective against both the active and dormant populations. Furthermore, persister control agents have the potential to be used in combination therapy with conventional antibiotics to improve the treatment of persistent infections. While conventional antibiotics kill normal cells, persister control agents can target the dormant cells. The capability to eradicate inactive cells can provide much-needed control of persistent infections. It also helps prevent patients from receiving continuous antibiotic treatment at sublethal levels to bacteria that can lead to resistance. Together, these findings emphasize the need for a change in antibiotic drug discovery from growth inhibition-based screening to rational approaches that target dormant cells.

## MATERIALS AND METHODS

### Chemical library

The SL#013 Gram Negative Antibacterials SDF file was obtained from the Antibacterial Library from Asinex Corp (73). All compounds in the screening library tested here are at least 92% pure based on liquid chromatography-mass spectrometry (LC-MS) analysis. The top three leads are at least 95% pure based on LC-MS analysis provided by Asinex Corp. No detectable impurities or secondary peaks were observed in the chromatograms (Fig. S1 to S3, S6 to S9, S12, S13, S16, S19 and S20). LC-MS data were obtained directly from Asinex Corp. and are listed in the supplementary document. NMR spectra were recorded with either a Bruker 800 MHz spectrometer or a Bruker 600 MHz spectrometer (both at the State University of New York—College of Environmental Science and Forestry) using DMSO-D6 (Sigma-Aldrich) as solvent. NMR spectra (^1^H and ^13^C) were recorded at room temperature (RT). Comprehensive characterization data and NMR spectra for all persister control agents are available in the supplementary document.

### Cheminformatic model

An integrative chemical-informatics workbench, ChemMine (33), developed by Backman et al., was used in this study. This workbench provides a web interface for a set of chemo-informatics and data mining tools that are useful for analyzing small molecules on the basis of structure, physicochemical similarities, and also customized data types (33). Using the SMILES string of the 80 chemical compounds obtained from the Asinex SL#013 Gram Negative Antibacterial Library from Asinex Corp. (73) and that of minocycline, eravacycline, and rifamycin SV obtained from the PubChem database, chemical properties such as logP (octanol-water partition), number of halogen groups, and number of aliphatic OH groups were extracted using JOELIB (32) within the ChemMine workbench. Raw data can be found in the supplemental material. Numeric data clustering, followed by the K-means sampling method, was executed to find compounds that have similar properties to those of minocycline, rifamycin SV, and eravacycline. Numerical data clustering was applied, which determines the distance of the data pair using Euclidean distance between the calculated properties (33). The unsupervised algorithm, K-means clustering, was applied to the processed data. It groups compounds based on similarity by minimizing the distance between individual data points and the cluster through calculations of centroids (74) (representing the theoretical multidimensional mean of all data points). Plots were generated within ChemMine using the CanvasXpress JavaScript library (33).

### Bacterial strains and growth media

*E. coli* HM22 (17, 40) (AT984 *dapA* zde-264::Tn10 *hipA7*), *E. coli* BW25113, *E. coli* BW25113 *ΔacrB* (49), *E. coli* BW25113 *ΔacrA* (49), *E. coli* BW25113 *ΔtolC* (49), *E. coli* BW25113 *ΔnagZ* (49) (JW1093), and UPEC ATCC53505, *Pseudomonas aeruginosa,* PAO1 (75) were routinely cultured in LB (76) containing 10 g/L NaCl, 5 g/L yeast extract, and 10 g/L tryptone. *E. coli* HM22 cultures were supplemented with 25 µg/mL diaminopimelic acid (DPA) (40) to ensure their ability to make new cell wall proteoglycan. Subcultures of UPEC ATCC53505 for persister isolation and biofilm formation were cultured in AUM (77, 78) as previously described. To make 1 L of AUM, two bulk solutions were made and autoclaved, followed by adding supplemented filtered solutions. Solution 1 contained 78.7 mM NaCl, 9 mM Na_2_SO_4_, 2.2 mM sodium citrate dihydrate, 0.1 mM Na_2_C_2_O_4_, 3.6 mM KH_2_PO_4_, and 21.5 mM KCl dissolved in 540 mL of water. Solution 2 contained 3 g of tryptic soy broth dissolved in 400 mL of water. After autoclaving solution 1 and solution 2, 3 mM CaCl_2_, 2 mM MgCl_2_, 15 mM NH_4_Cl, 6 mM creatine, and 200 mM of urea were added. The pH of the solution was adjusted to 6.3.

### Viability assay

Normal and persister cells were treated with each chemical compound based on a previously reported protocol (17). Briefly, an overnight culture of *E. coli* HM22 was sub-cultured in LB supplemented with DPA with a starting OD_600_ of 0.05. When the cultures reached mid-exponential phase (OD_600_ 0.3–0.45), cells were collected by centrifugation at 13,000 rpm for 3 min at room temperature. The cells were washed three times with PBS and then resuspended in 500 µL of PBS with the cell density adjusted to an OD_600_ of 0.5. For the normal population, the cells in PBS were immediately treated with the chemical compound of interest for 1 h at 37°C with shaking at 200 rpm. After 1 h, the treated samples were collected by centrifugation (3 min at 13,000 rpm) and washed three times with PBS to remove the remaining free compound of testing in the solution. The cells were then resuspended in PBS and plated on LB agar plates to count CFU using the drop plate method (79). Relative viability was determined by normalizing the CFU by the untreated control. Each experimental condition was tested with three biological replicates.

To isolate persister cells, the mid-exponential phase cells were treated with 100 µg/mL of ampicillin (Sigma-Aldrich) for 3.5 h at 37°C with shaking at 200 rpm in LB to kill normal cells as reported previously (17). After isolation, the cells were washed three times with PBS to remove extracellular antibiotics and then proceeded to chemical compound treatment in PBS at 37°C as mentioned above. Each experimental condition was tested with three biological replicates. To evaluate the effects of the chemical compounds on efflux pump mutants, *E. coli* BW25113 and its isogenic mutants of *ΔacrB, ΔacrA, and ΔtolC* were treated in the same way as described above.

### Accumulation assay

The intracellular concentration of each selected compound was quantified by following a previously described protocol (17). Briefly, the reporter strain *E. coli* BW25113 *ΔtolC* was treated with *E. coli* HM22 persister cell lysate spiked with known concentrations of a chosen chemical compound to generate a standard curve (Fig. S25). This was then used to determine the concentration of the compound in unknown samples. Cell lysates from treated *E. coli* HM22 persister cells and untreated controls were extracted using chloroform after treatment as previously described (17, 80). Then the cell debris was separated and discarded after centrifugation at 10,000 rpm for 5 min. The samples were concentrated five times and then evaporated overnight in a vacuum desiccator. The intracellular compound was dissolved in 100 mL sterile PBS by vortexing for 5 min. Next, the samples were used to treat the reporter strain in 500 µL of PBS with an OD_600_ of 0.5. After 1 h of incubation, the treated samples were collected by centrifugation and washed three times with PBS to remove the remaining free compound. The cells were then resuspended in PBS and plated on LB agar plates to count CFU using the drop plate method (79). Antibiotic concentration was determined by fitting the standard curve (Fig. S25). Individual cell volume of *E. coli* HM22 persisters was calculated based on microscopic images. The total cell numbers were determined using a hemocytometer.

### Amphiphilic moment and globularity calculations

To produce 3D structures, LigPrep (Maestro software, Schrödinger Release 2023-1**,** LLC, New York, NY 2023) using OPLS4 force fields was used. Tautomeric and protonation states were determined using Epik (Schrödinger Release 2023-1**,** LLC, New York, NY 2023) at pH 7.4. When generating the 3D structures of the chemical compounds through LigPrep, various forms are considered, including tautomer, protonation states, and stereoisomers, which are identified as structural variants. The amphiphilic moment was calculated based on the 3D structures using the amphiphilic calculator within the Maestro software. Globularity was calculated using QikPrep (Schrödinger Release 2023-1**,** LLC, New York, NY 2023).

### Isolation of UPEC persister cells

Briefly, an overnight culture of UPEC was sub-cultured in AUM with a starting OD_600_ of 0.05. When the cultures reached mid-exponential phase (OD_600_ 0.3–0.45), 100 µg/mL of ampicillin or 1 µg/mL of ciprofloxacin hydrochloride (Alfa Aesar) was added and incubated for 3.5 h at 37°C with shaking at 200 rpm in AUM to kill normal cells. Concentration for persister isolation was based on previously reported studies (17, 81, 82). A time-dependent killing curve was generated to confirm persister isolation (Fig. S25). After isolation, the persister cells were washed three times with PBS to remove extracellular antibiotics and then proceeded to chemical compound treatment as mentioned above in the viability assay section.

### Growth curve of resuscitated persister cells

Persister cells were washed and resuspended in 200 µL LB to an OD_600_ of 0.2. The samples were then transferred to a 96-well plate and incubated at 37°C with shaking at 200 rpm. Growth was monitored at OD_600_ nm for 32 h using an Epoch 2 Microplate Spectrophotometer (BioTek, Winooski, VT, USA).

### Isolation of PAO1 persister cells

Briefly, an overnight culture of PAO1 was treated with 200 µg/mL of CCCP (MedChem Express) for 3.5 h at 37°C with shaking at 200 rpm in LB. CCCP concentration for persister isolation was based on previously reported studies (55, 56). After isolation, the cells were washed three times with PBS to remove extracellular CCCP and then proceeded to chemical compound treatment as mentioned above.

### Biofilm growth

Biofilms were grown on polydimethylsiloxane (PDMS) surfaces. Using the Sylgard 184 elastomer kit, a 10:1 (wt/wt) of base: curing agent was poured, mixed, and vacuumed in a 50 mL Falcon tube. The PDMS was polymerized at 60°C for 24 h on a 25 cm petri plate. PDMS surfaces of 10 mm × 0.5 mm were cut out and then sterilized under UV light for 1 h before use. Sterilized PDMS surfaces were transferred into a new petri dish (four to six surfaces per 10 cm plate) for biofilm formation. Briefly, an overnight culture of UPEC (~16 h) was used to inoculate the biofilm culture with a starting density of OD_600_ of 0.05 in 5 mL AUM. The biofilms were grown for 48 h at 37°C. Then, each PDMS surface with biofilm was washed three times with PBS, transferred to a new petri dish with 100 µg/mL of compound 173 or 175 in PBS, and treated for 1 h at 37°C. After treatment, the biofilm viability was determined by staining and imaging or CFU count. For image analysis, each PDMS surface was stained with LIVE/DEAD BacLight bacterial viability kit (Life Technologies Inc., Carlsbad, CA, USA) in 0.85% NaCl solution for 15 min in the dark. The stained biofilm samples were imaged using an Axio Imager M1 fluorescence microscope (Carl Zeiss Inc., Berlin, Germany) with an Orca-Flash 4.0 LT camera (Hamamatsu Photonics, Hamamatsu City, Japan). Biomass analysis was quantified using COMSTAT (83–85). To determine CFU, each treated PDMS surface was transferred to a 5 mL polystyrene tube containing 1 mL of PBS. The tubes containing the treated samples were gently sonicated for 1 min using a Branson Tabletop Ultrasonic (Model B200, Brookfield, CT, USA) and vortexed for 30 s to detach biofilm cells from the PDMS surface (80, 86). The solution in the tubes was used to determine biofilm viability based on CFU.

### Quantification of persister levels in treated biofilm

Detached biofilm cells were treated with 100 µg/mL of AMP in AUM medium. The biofilms were treated for 3.5 h at 37°C. After treatment, the biofilm viability was determined by counting CFU as described above.

### Effects of the *nagZ* mutation

To test if N-acetylglucosaminidase plays a role in *E. coli* killing by selected compounds, the *nagZ* mutant was obtained from the Keio collection (49) and compared with the wild-type strain BW25113 for the effects of selected compounds. The same protocol for HM22 normal and persister tests was followed.

### Minimum inhibitory concentration testing

An overnight culture of *E. coli* HM22 was used to inoculate LB medium in 96-well plates with a starting OD_600_ of 0.01. Each compound was tested at 0, 2, 4, 8, 16, 32, 64, and 128 µg/mL. The cultures were incubated at 37°C with shaking at 200 rpm for 24 h. The presence/absence of growth was used to determine MIC.

### Live/dead cell population monitoring with flow cytometry

Isolated *E. coli* HM22 persister cells were treated with 100 µg/mL of Asinex compounds 161, 169, and 175 for 1 h at 37°C with shaking at 200 rpm. The cells were then washed three times with PBS, resuspended in 200 µL LB medium, and incubated at 37°C with shaking at 200 rpm. At each time point, 0, 1, 2, 3, and 14 h, the cells were centrifuged, resuspended in PBS, and stained with LIVE/DEAD BacLight bacterial viability kit (Life Technologies Inc., Carlsbad, CA, USA). GFP and PI fluorescence data were collected using a BD Accuri C6 Plus flow cytometer.

### Statistical analysis

Error bars in all figures represent the standard error of the mean. All data were analyzed using ANOVA followed by the Tukey test using SAS version 9.13 (SAS Institute, Cary, NC, USA). Differences with *P* < 0.05 were statistically significant (* *P*-value ≤ 0.05, ** *P*-value ≤ 0.01, *** *P*-value ≤ 0.001, and ****P-value ≤ 0.0001).

## SUPPLEMENTAL MATERIAL

Supplemental material aac.01814-24-s0001.docx Table S1; Fig. S1 to S30.

Supplemental data aac.01814-24-s0002.xlsx Chemical compound properties.

ASM does not own the copyrights to Supplemental Material that may be linked to, or accessed through, an article. The authors have granted ASM a non-exclusive, world-wide license to publish the Supplemental Material files. Please contact the corresponding author directly for reuse.
