# Supplementary material for: A rational approach to discovering new persister control agents
Source: Antimicrob Agents Chemother. 2025 Jul 31;69(9):e01814-24. doi: 10.1128/aac.01814-24 (PMC12406675; doi:10.1128/aac.01814-24)
Supplement: Supplemental material — Table S1; Fig. S1 to S30. [file aac.01814-24-s0001.docx]

**Supplemental Material**

**A rational approach to discovering new persister control agents**

Sweta Roy^1^, Zeynep S. Cakmak^1^, Sheila Priscilla Kyeremeh^1^, Shikha Nangia^1^, Juntao Luo^2^, and Dacheng Ren^1,3,4*^

^1^Department of Biomedical and Chemical Engineering, Syracuse University, Syracuse, NY 13244, USA

^2^Department of Pharmacology, State University of New York Upstate Medical University, Syracuse, NY 13210, USA

^3^Department of Civil and Environmental Engineering, Syracuse University, Syracuse, NY 13244, USA

^4^Department of Biology, Syracuse University, Syracuse, NY 13244, USA

***Corresponding author:** Dacheng Ren: Phone +1-315-443-4409. Fax +1-315-443-9175.

**Email:** [dren@syr.edu](mailto:dren@syr.edu)

**Keywords:** persister, killing, drug discovery, chemoinformatic, clustering

**Table S1.** **List of compound numbers and their corresponding Asinex ID.** This table lists the chemical compound numbers along with their corresponding Asinex identification numbers for each compound tested in this study.

| **Chemical Compound ID** | **ASINEX ID Number** |
| --- | --- |
| 100 | LAS_22461675 |
| 101 | LAS_32135590 |
| 102 | LAS_34000090 |
| 103 | LAS_34036730 |
| 104 | LAS_51136079 |
| 105 | LAS_51177972 |
| 106 | LAS_51380924 |
| 107 | LAS_51382001 |
| 108 | LAS_51635358 |
| 109 | LAS_51635707 |
| 110 | LAS_52154070 |
| 111 | LAS_52154080 |
| 112 | LAS_52154082 |
| 113 | LAS_52154104 |
| 114 | LAS_52154116 |
| 115 | LAS_52154128 |
| 116 | LAS_52154130 |
| 117 | LAS_52154152 |
| 118 | LAS_52154154 |
| 119 | LAS_52154184 |
| 120 | LAS_52154194 |
| 121 | LAS_52157595 |
| 122 | LAS_52157601 |
| 123 | LAS_52157603 |
| 124 | LAS_52157607 |
| 125 | LAS_52157609 |
| 126 | LAS_52157613 |
| 127 | LAS_52157615 |
| 128 | LAS_52160841 |
| 129 | LAS_52160851 |
| 130 | LAS_52160863 |
| 131 | LAS_52160873 |
| 132 | LAS_52160881 |
| 133 | LAS_52160913 |
| 134 | LAS_52160923 |
| 135 | LAS_52160933 |
| 136 | LAS_52160943 |
| 137 | LAS_52160953 |
| 138 | LAS_52160961 |
| 139 | LAS_52161003 |
| 140 | LAS_52161013 |
| 141 | LAS_52171209 |
| 142 | LAS_52171221 |
| 143 | LAS_52171223 |
| 144 | LAS_52171241 |
| 145 | LAS_52171265 |
| 146 | LAS_52171288 |
| 147 | LAS_52171786 |
| 148 | LAS_52171806 |
| 149 | LAS_52171812 |
| 150 | LAS_52171814 |
| 151 | LAS_52171828 |
| 152 | LAS_52171830 |
| 153 | LAS_52171836 |
| 154 | LAS_52171840 |
| 155 | LAS_52202332 |
| 156 | LAS_52202336 |
| 157 | LAS_52451048 |
| 158 | LAS_52451118 |
| 159 | LAS_52463552 |
| 160 | LAS_52463611 |
| 161 | LAS_52466748 |
| 162 | LAS_52475652 |
| 163 | LAS_52475658 |
| 164 | LAS_52475715 |
| 165 | LAS_52480643 |
| 166 | LAS_52480647 |
| 167 | LAS_52481503 |
| 168 | LAS_52483389 |
| 169 | LAS_52485299 |
| 170 | LAS_52485311 |
| 171 | LAS_52503902 |
| 172 | LAS_52504673 |
| 173 | LAS_52504712 |
| 174 | LAS_52504731 |
| 175 | LAS_52505571 |
| 176 | LAS_52505624 |
| 177 | LAS_52505630 |
| 178 | LAS_52506179 |
| 179 | LAS_52506188 |


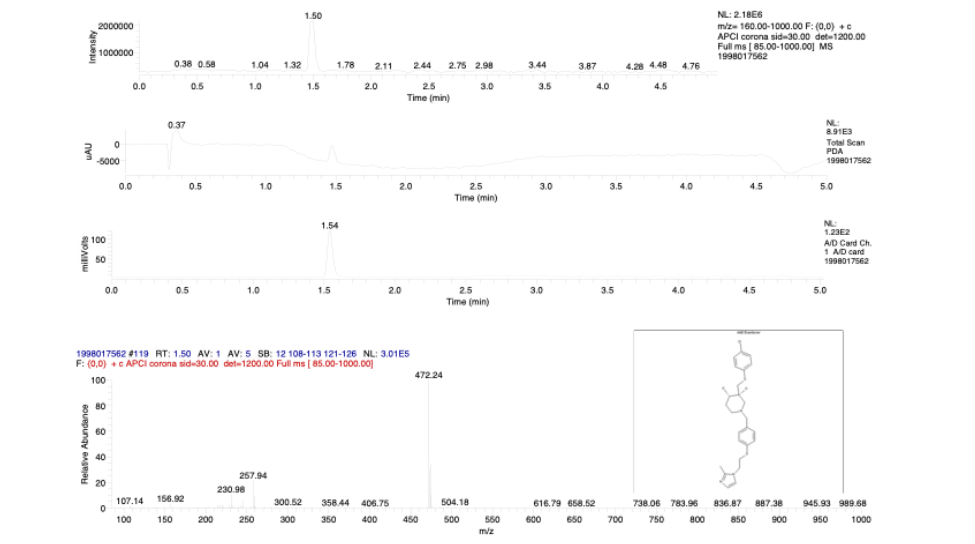


**Figure S1.** LC-MS analysis of compound **159**.


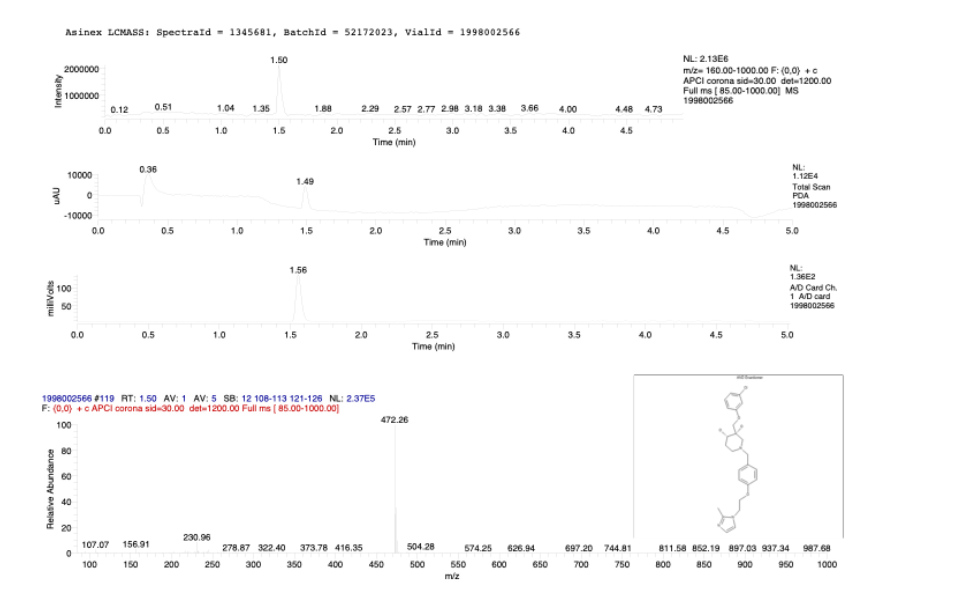


**Figure S2.** LC-MS analysis of compound **160**.


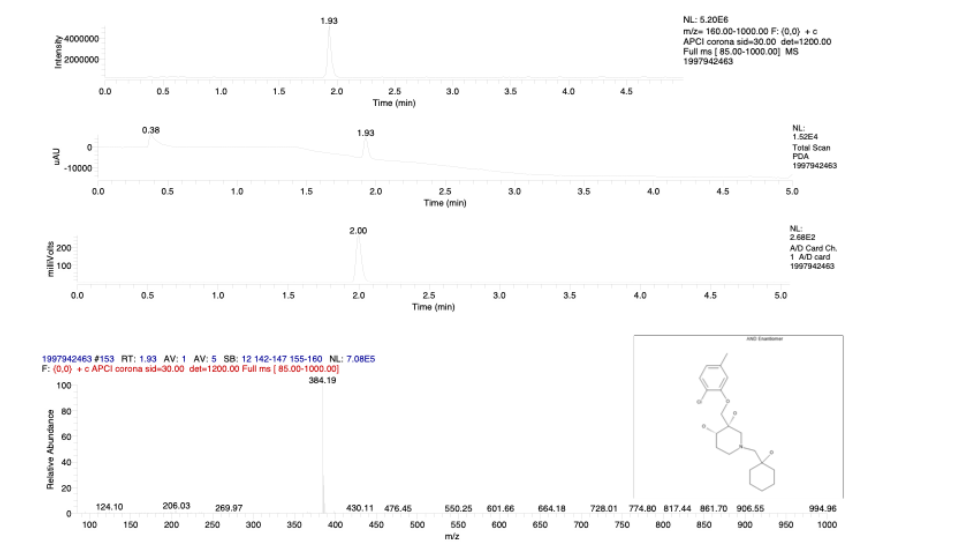


**Figure S3.** LC-MS analysis of compound **161**.


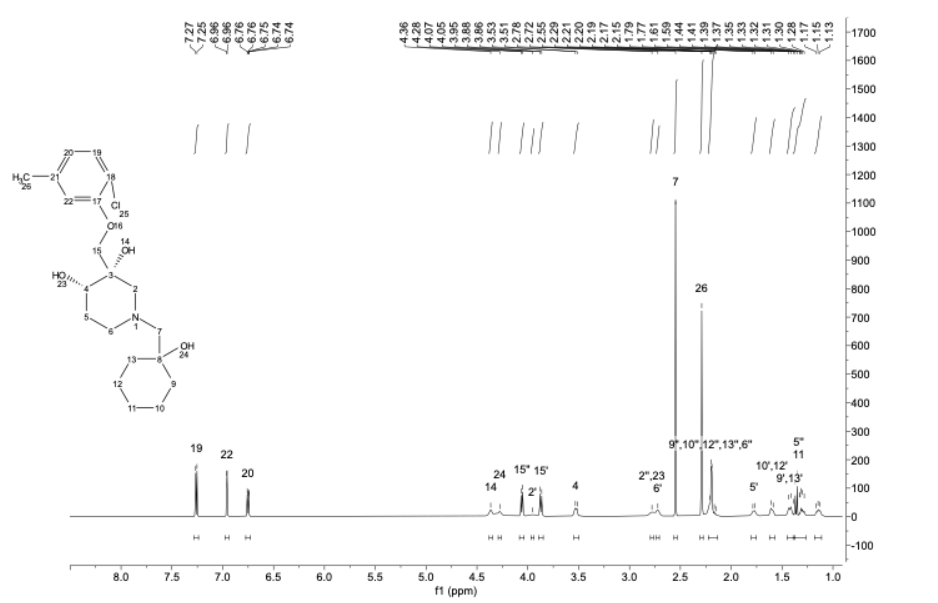


**Figure S4.** ^1^H NMR (600 MHz, DMSO) of compound **161.** δ 7.26 (d, *J* = 8.0 Hz, 1H), 6.96 (d, *J* = 1.9 Hz, 1H), 6.77 – 6.73 (m, 1H), 4.36 (s, 1H), 4.28 (s, 1H), 4.06 (d, *J* = 9.3 Hz, 1H), 3.95 (s, 1H), 3.87 (d, *J* = 9.3 Hz, 1H), 3.52 (d, *J* = 10.4 Hz, 1H), 2.78 (s, 1H), 2.72 (s, 1H), 2.55 (s, 3H), 2.29 (s, 3H), 2.20 (t, *J* = 6.1 Hz, 4H), 1.78 (d, *J* = 11.2 Hz, 1H), 1.60 (d, *J* = 13.1 Hz, 1H), 1.42 (d, *J* = 13.4 Hz, 2H), 1.39 – 1.27 (m, 2H), 1.15 (t, *J* = 12.0 Hz, 1H).


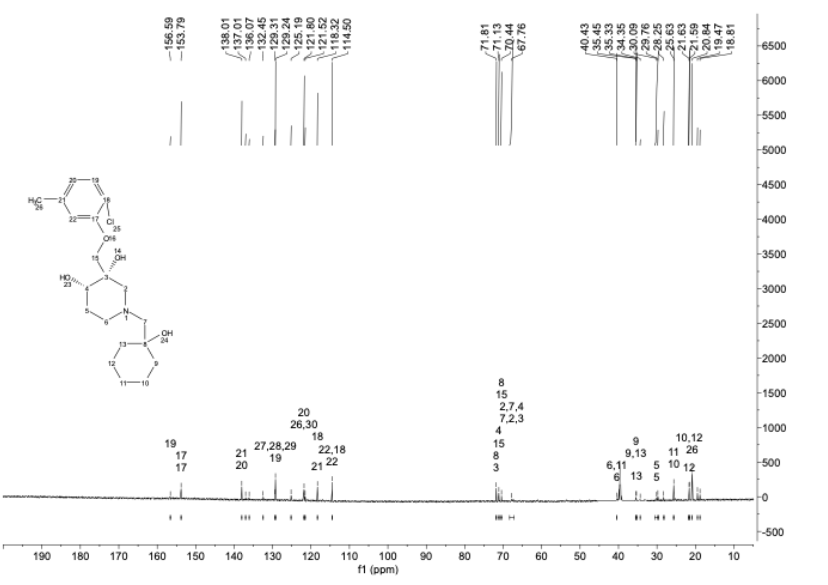


**Figure S5.** ^13^C NMR (201 MHz, DMSO) of compound **161**. δ 156.59, 153.79, 138.01, 137.01, 136.07, 132.45, 129.31, 129.24, 125.19, 121.80, 121.52, 118.32, 114.50, 71.81, 71.13, 70.44, 67.76, 40.43, 35.45, 35.33, 34.35, 30.09, 29.76, 28.25, 25.63, 21.63, 21.59, 20.84, 19.47, 18.81.


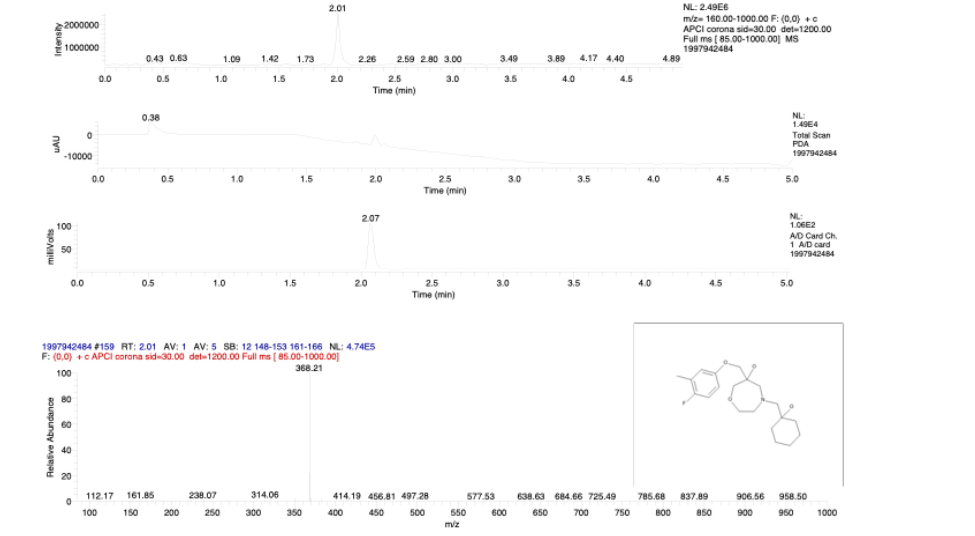


**Figure S6.** LC-MS analysis of compound **165**.


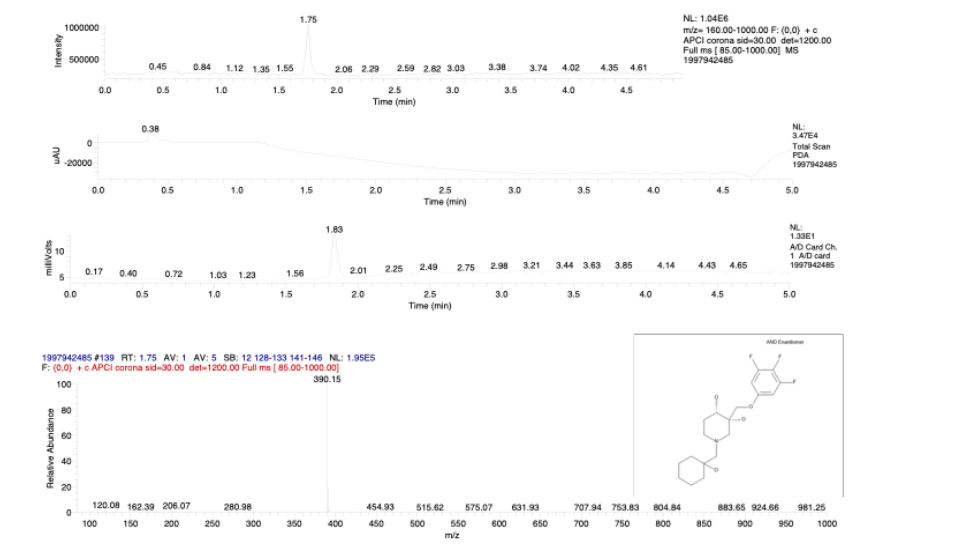


**Figure S7.** LC-MS analysis of compound **166**.


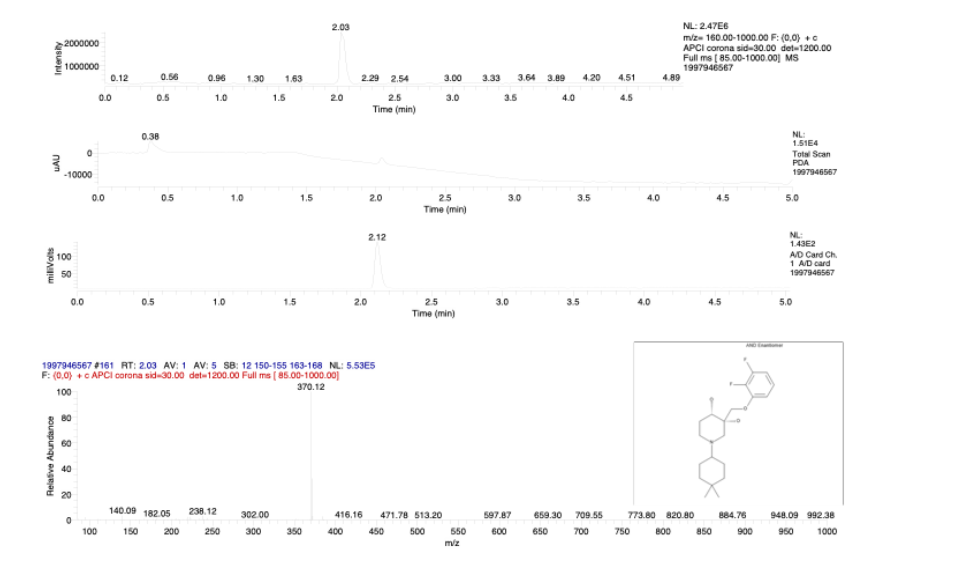


**Figure S8.** LC-MS analysis of compound **167**.


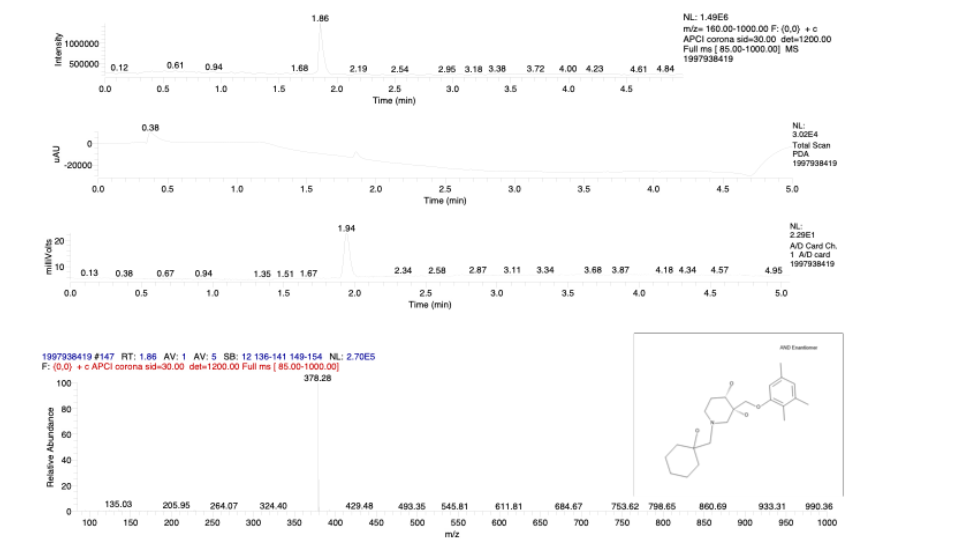


**Figure S9.** LC-MS analysis of compound **169**.


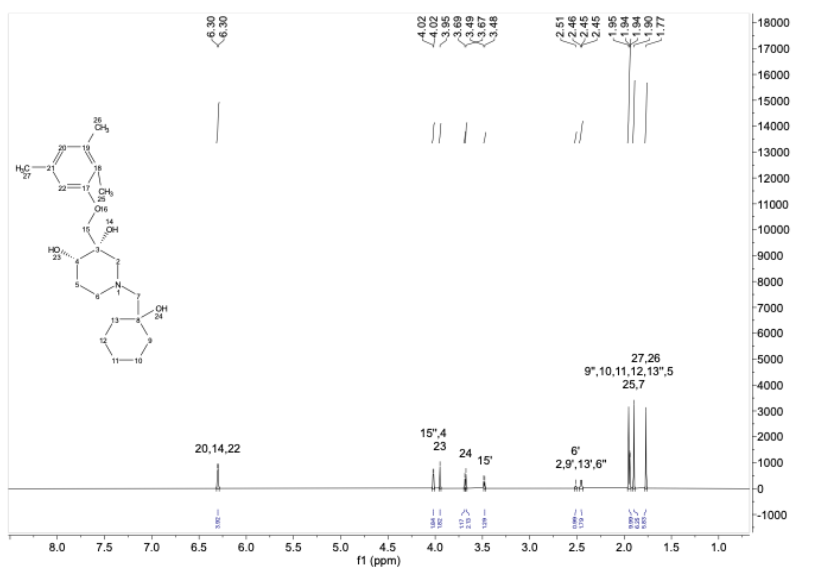


**Figure S10.** ^1^H NMR (800 MHz, DMSO) of compound **169**. δ 6.30 (d, *J* = 4.6 Hz, 4H), 4.02 (d, *J* = 6.5 Hz, 2H), 3.95 (s, 2H), 3.69 (s, 1H), 3.67 (s, 2H), 3.48 (s, 1H), 2.51 (s, 1H), 2.47 – 2.44 (m, 2H), 1.96 – 1.93 (m, 9H), 1.90 (s, 6H), 1.77 (s, 5H).


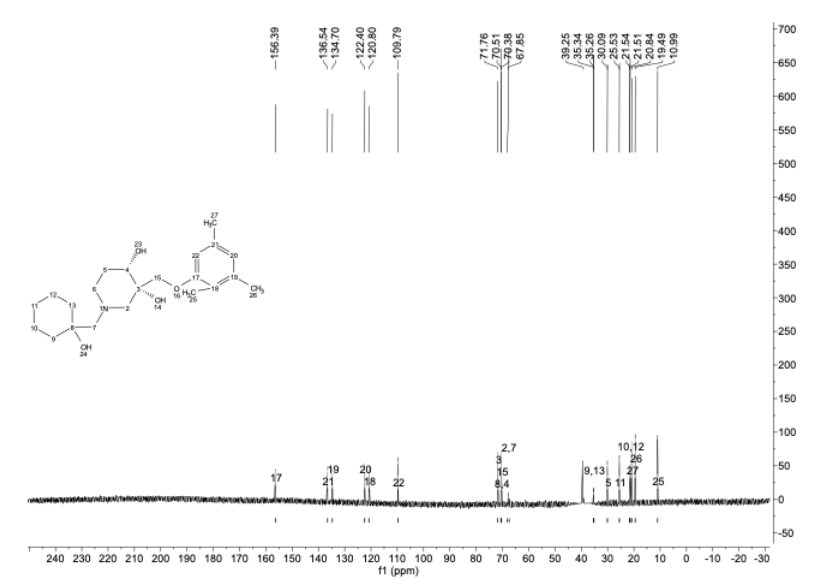


**Figure S11. ^1^**^3^C NMR (201 MHz, DMSO) of compound **169**. δ 156.39, 136.54, 134.70, 122.40, 120.80, 109.79, 71.76, 70.51, 70.38, 67.85, 35.34, 35.26, 30.09, 25.53, 21.54, 21.51, 20.84, 19.49, 10.99.


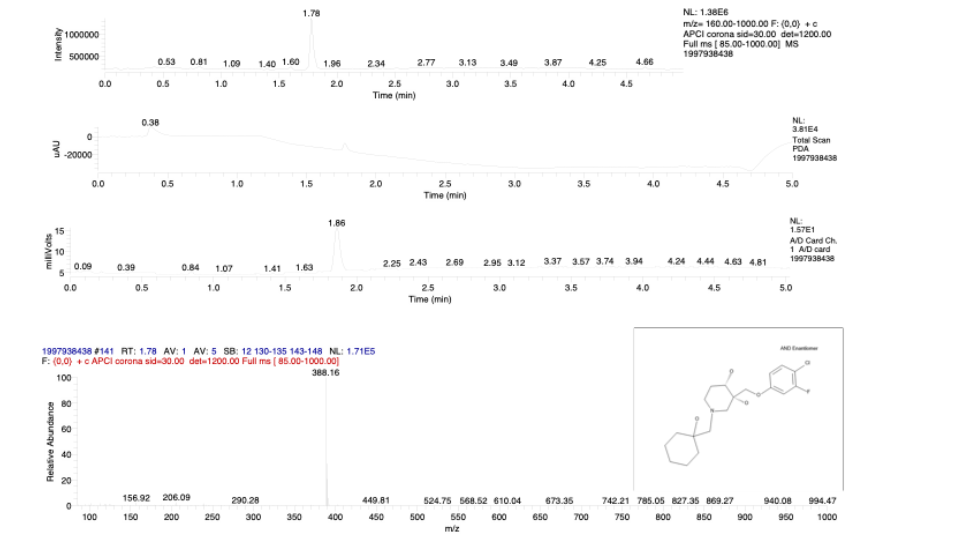


**Figure S12.** LC-MS analysis of compound **170**.


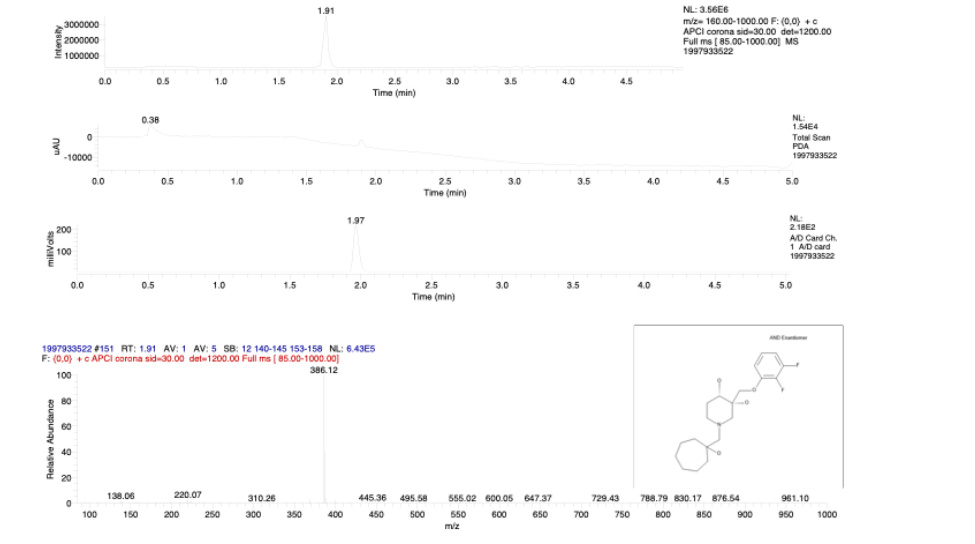


**Figure S13.** LC-MS analysis of compound **171**.


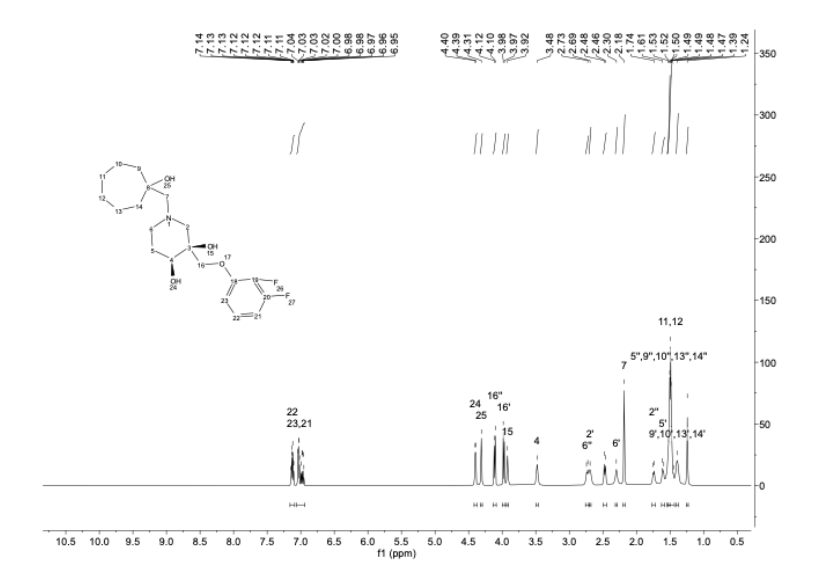


**Figure S14.** ^1^H NMR (600 MHz, DMSO) of compound **171**. δ 7.12 (ddd, *J* = 8.4, 6.2, 2.1 Hz, 1H), 7.06 – 6.94 (m, 2H), 4.40 (d, *J* = 6.1 Hz, 1H), 4.31 (s, 1H), 4.11 (d, *J* = 9.6 Hz, 1H), 3.98 (d, *J* = 9.6 Hz, 1H), 3.92 (s, 1H), 3.48 (s, 1H), 2.73 (d, *J* = 11.1 Hz, 1H), 2.69 (s, 1H), 2.47 (d, *J* = 11.3 Hz, 1H), 2.30 (s, 1H), 2.18 (s, 2H), 1.74 (d, *J* = 10.5 Hz, 1H), 1.60 (d, *J* = 10.8 Hz, 1H), 1.52 (s, 4H), 1.54 – 1.43 (m, 5H), 1.39 (s, 2H), 1.24 (s, 1H).


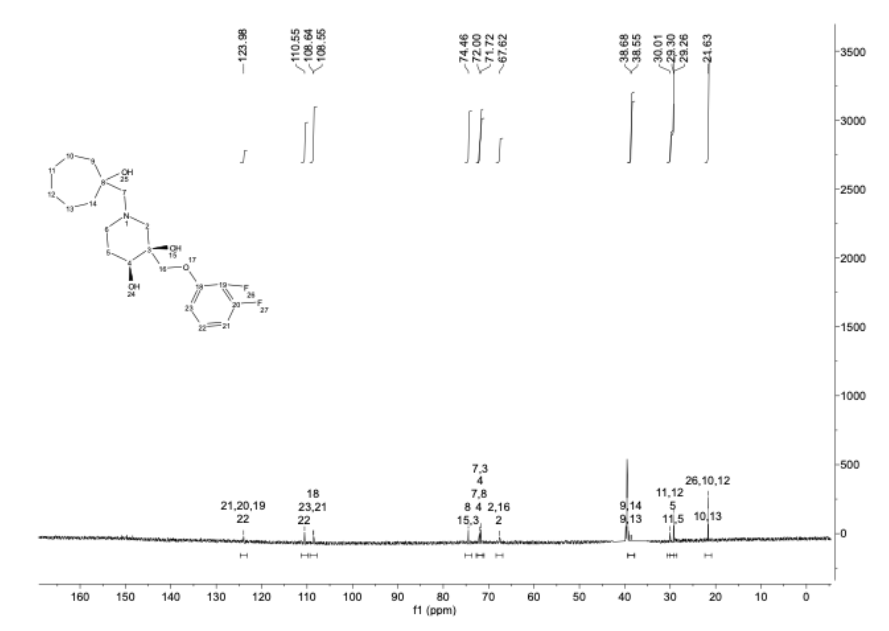


**Figure S15.** ^13^C NMR (201 MHz, DMSO) of compound **171**. δ 124.58, 111.15, 109.19 (d, *J* = 17.5 Hz), 75.06, 72.60, 72.32, 68.22, 39.28, 39.15, 30.61, 29.88 (d, *J* = 7.6 Hz), 22.23.


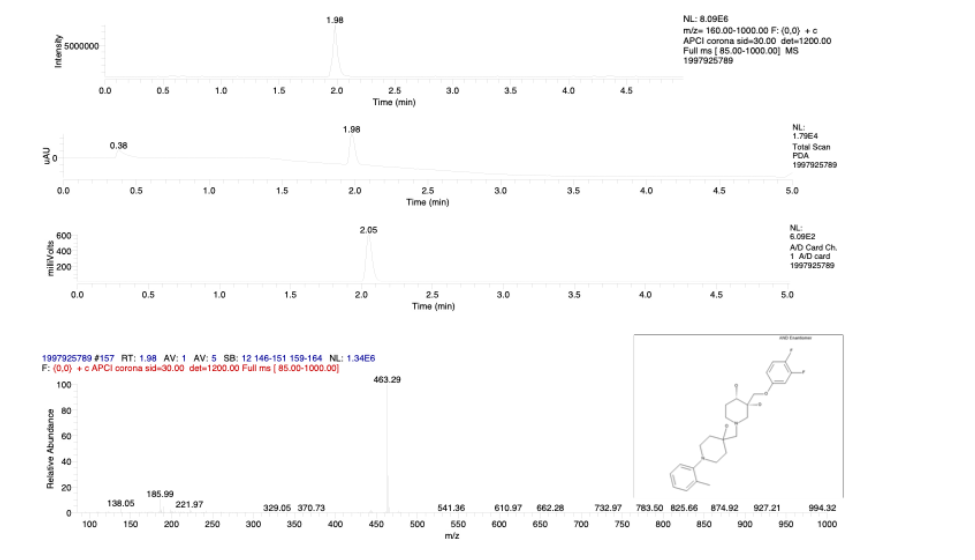


**Figure S16.** LC-MS analysis of compound **173**.


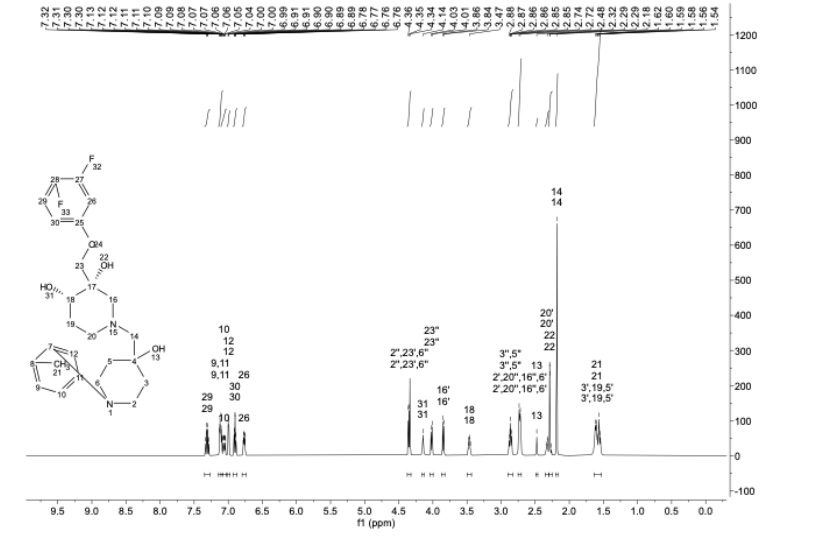


**Figure S17.** ^1^H NMR (600 MHz, DMSO) of compound **173**. δ 7.31 (dt, *J* = 10.6, 9.3 Hz, 1H), 7.11 (ddd, *J* = 10.8, 6.0, 3.8 Hz, 2H), 7.11 – 7.02 (m, 1H), 7.02 – 6.97 (m, 1H), 6.90 (td, *J* = 7.4, 1.2 Hz, 1H), 6.77 (ddt, *J* = 9.3, 3.4, 1.7 Hz, 1H), 4.37 – 4.32 (m, 2H), 4.14 (s, 1H), 4.02 (d, *J* = 9.5 Hz, 1H), 3.85 (d, *J* = 9.5 Hz, 1H), 3.47 (dt, *J* = 9.9, 5.3 Hz, 1H), 2.90 – 2.83 (m, 2H), 2.73 (d, *J* = 10.4 Hz, 4H), 2.48 (s, 0H), 2.33 (d, *J* = 10.2 Hz, 1H), 2.29 (d, *J* = 1.5 Hz, 2H), 2.18 (s, 3H), 1.64 – 1.53 (m, 6H).


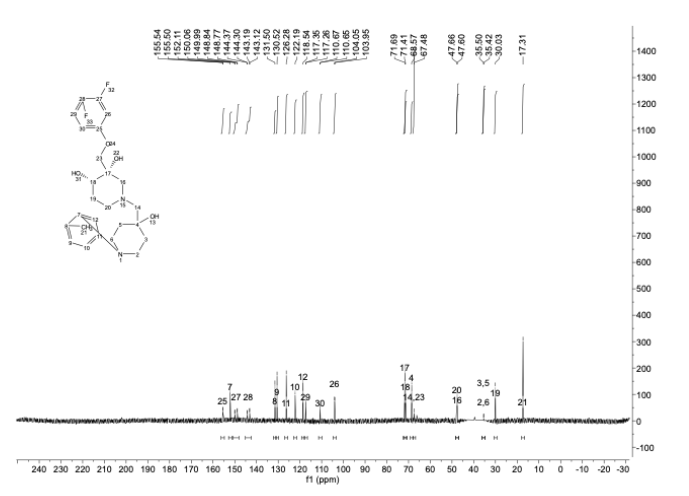


**Figure S18.** ^13^C NMR (201 MHz, DMSO) of compound **173.** δ 155.62 (d, *J* = 8.6 Hz), 152.21, 149.52 (dd, *J* = 244.4, 13.4 Hz), 143.85 (dd, *J* = 236.9, 13.0 Hz), 131.60, 130.62, 126.39, 122.30, 118.65, 117.41 (d, *J* = 18.2 Hz), 110.77 (d, *J* = 4.1 Hz), 104.11 (d, *J* = 20.2 Hz), 71.80, 71.51, 68.67, 67.59, 47.76, 47.70, 35.61, 35.52, 30.13, 17.42.


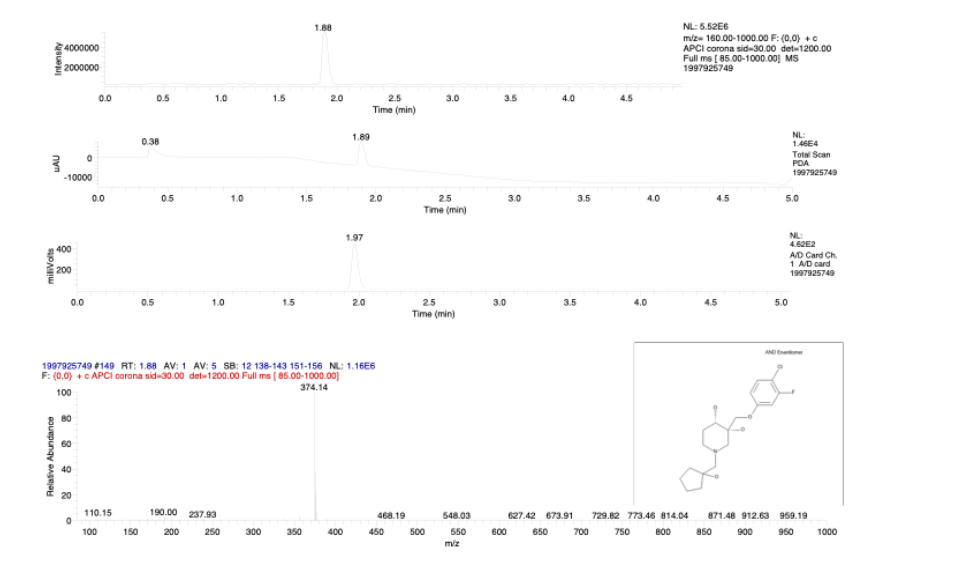


**Figure S19.** LC-MS analysis of compound **174**.
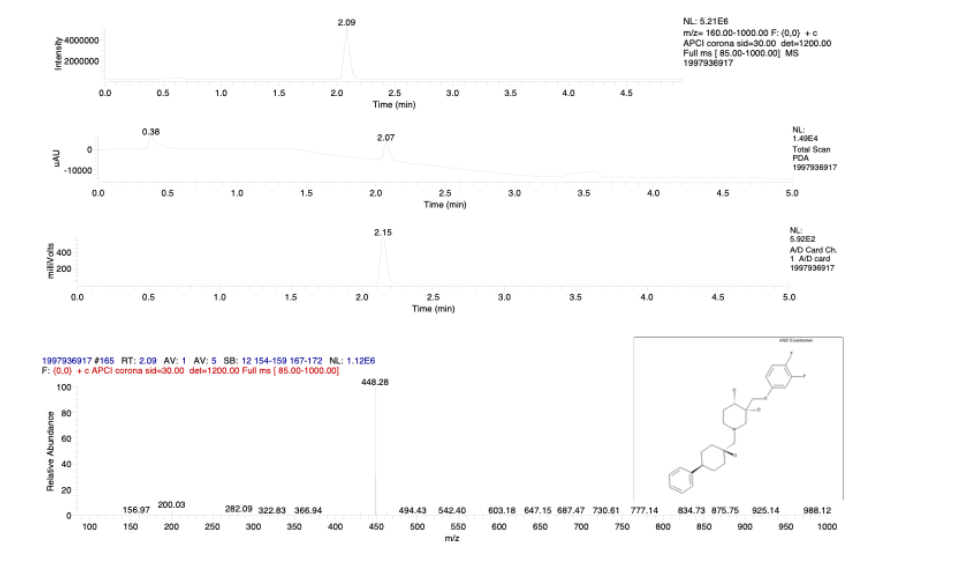


**Figure S20.** LC-MS analysis of compound **175**.


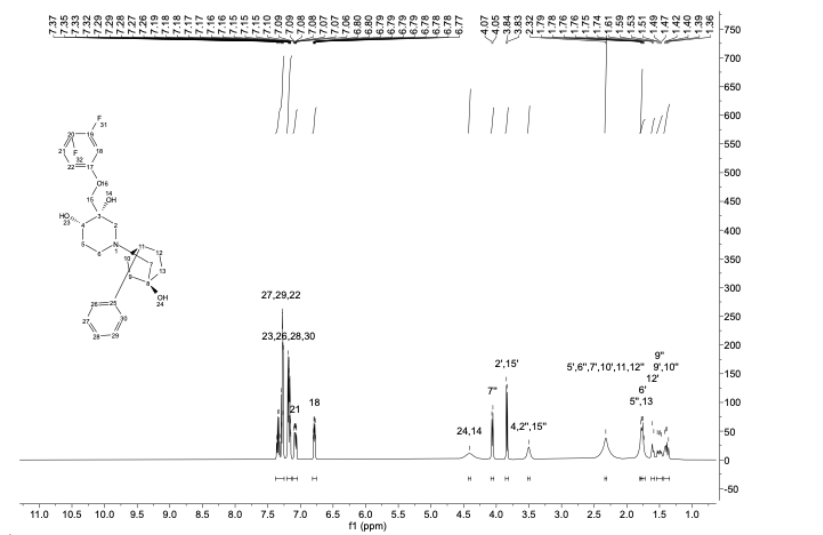


**Figure S21**. ^1^H NMR (600 MHz, DMSO) of compound **175**. δ 7.38 – 7.25 (m, 4H), 7.21 – 7.13 (m, 4H), 7.08 (ddd, *J* = 12.7, 6.7, 3.0 Hz, 1H), 6.79 (ddt, *J* = 10.5, 4.6, 2.3 Hz, 1H), 4.41 (s, 2H), 4.06 (d, *J* = 9.5 Hz, 1H), 3.84 (d, *J* = 9.4 Hz, 1H), 3.50 (s, 1H), 2.32 (s, 5H), 1.78 (s, 3H), 1.80 – 1.72 (m, 1H), 1.60 (d, *J* = 13.4 Hz, 1H), 1.50 (dd, *J* = 26.6, 11.7 Hz, 1H), 1.39 (dd, *J* = 21.9, 11.5 Hz, 1H).


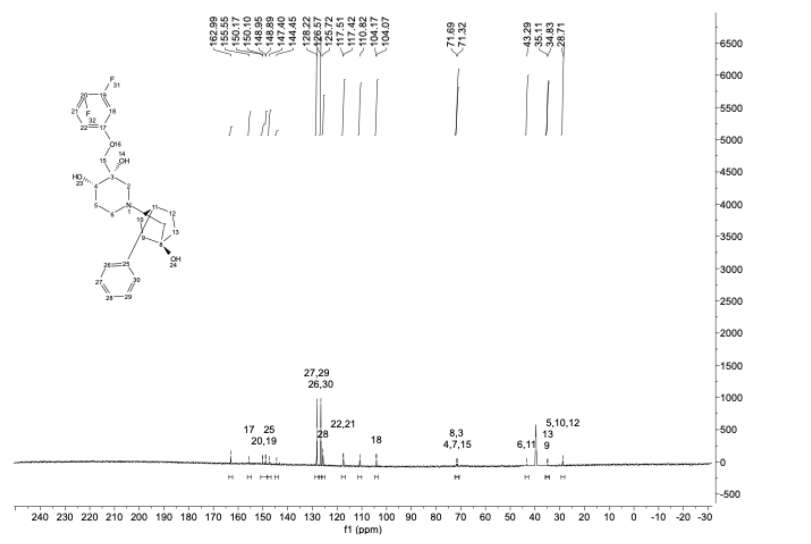


**Figure S22.** ^13^C NMR (201 MHz, DMSO) of compound **175**. δ 162.99, 155.55, 149.53 (dd, *J* = 244.6, 13.6 Hz), 147.40, 144.45, 128.22, 126.57, 125.72, 117.46 (d, *J* = 18.2 Hz), 110.82, 104.12 (d, *J* = 20.1 Hz), 71.69, 71.32, 43.29, 35.11, 34.83, 28.71.

**Figure S23. Validation of persister isolation method for *E. coli* HM22, UPEC and PAO1.** **(a)** *E. coli* HM22 were grown to exponential phase and treated with different concentrations of AMP (from 0-120 µg/mL) for 3.5 h. Viability was determined based on CFU after plating. **(b)** Stationary phase PAO1 were treated with different concentrations of CCCP (from 0-200 µg/mL) for 3 h. Viability was determined based on CFU after plating. UPEC was grown in exponential phase and treated with **(c)** 100 µg/mL of AMP or **(d)** 1 µg/mL of CIP for a total of 7 h in AUM. At each time point, the cells were washed, and viability was determined via CFU count after plating in LB plates (black dots) or AUM plates (blue dots).

**Figure S24.** **Evaluating the negative controls**. Six randomly chosen compounds (106, 108, 121, 126, 127, 147) from the cluster group (screening results to the left) that did not cluster with eravacycline, minocycline and rifamycin SV was evaluated for killing against *E. coli* HM22 normal and persister cells at a concentration of 100 g/mL (bar graphs to the right). The SMILES of each of the six compounds are shown in the table.

**Figure S25.** **Standard curve to calculate intracellular concentrations.** Standard curves were generated to assess the intracellular levels of compounds 161, 169, 171, 173, and 175. These curves were obtained by measuring the killing activity of each compound against *E. coli* BW25113 *ΔtolC* reporter strain using lysates from either normal or persister of *E. coli* HM22 cells spiked with known concentrations of the respective compounds.

**Figure S26. Growth curve of treated UPEC persister cells during wake up in different media.** The graph shows resuscitation of AMP UPEC persisters (left) and CIP UPEC persisters (right) in LB (black) and AUM (light blue). The growth of each type of persister cells in different media was monitored based on OD_600nm_ over 32 h.


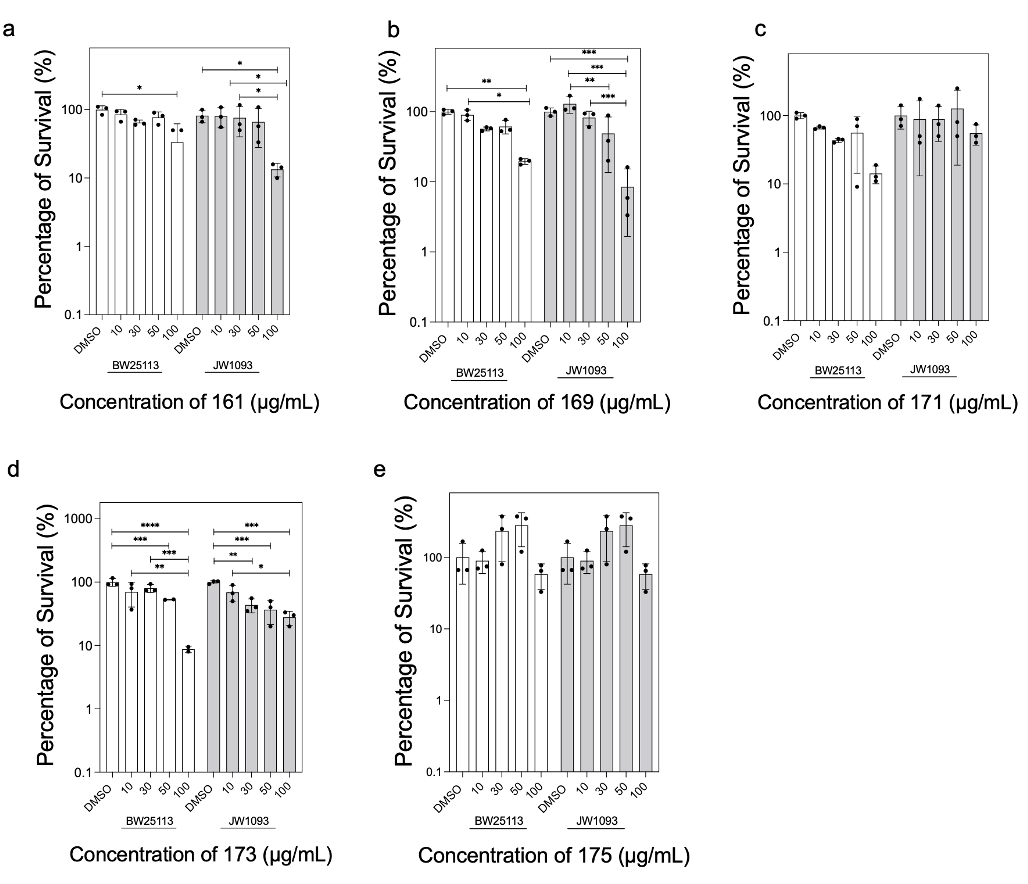


**Figure S27.** **Effects of selected compounds on normal cells of *E. coli* BW25113 and its *nagZ* mutant (JW1093).** Compounds 161, 169, 171, 173, and 175 were tested on *E. coli* BW25113 wild-type (white bars) and the *nagZ* mutant (grey bars). CFU counts were determined and normalized to the untreated control for each condition. Treatment was performed in PBS for 1 h. Data represent means ± SE (n = 3).


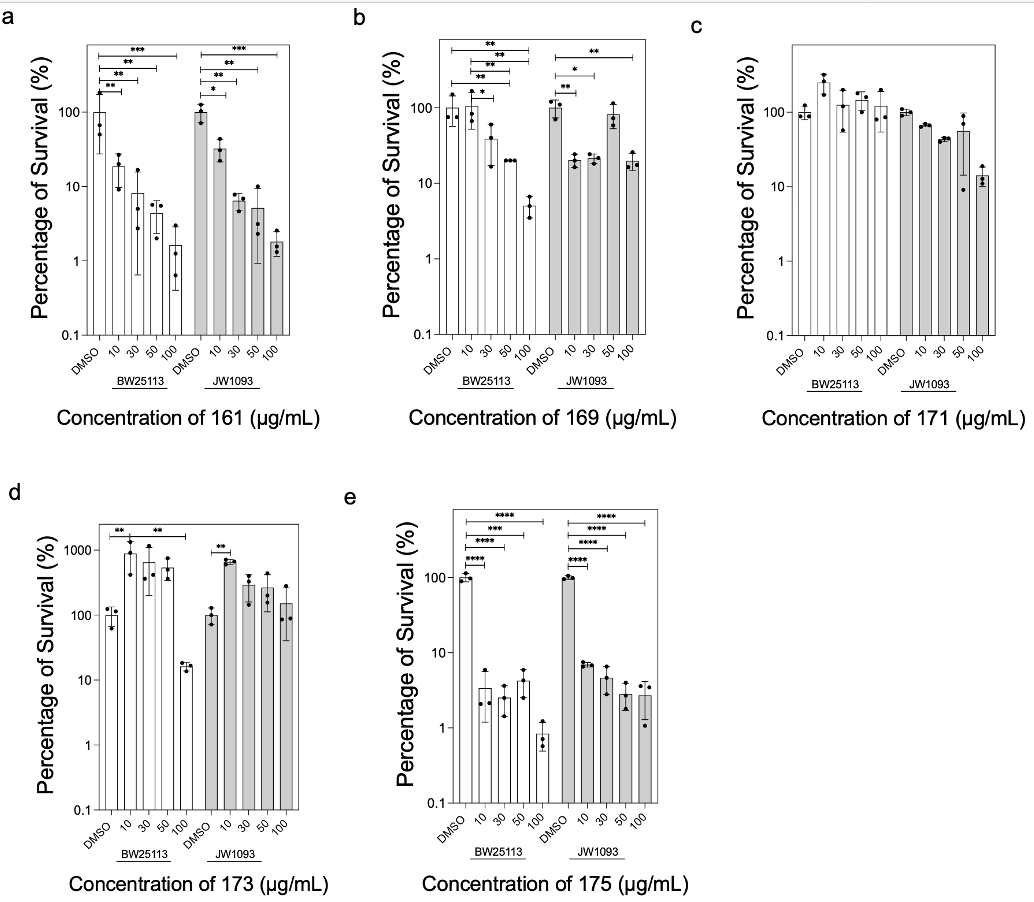


**Figure S28.** **Effects of selected compounds on persister cells of *E. coli* BW25113 and its *nagZ* mutant (JW1093).** Compounds 161, 169, 171, 173, and 175 were tested on *E. coli* BW25113 wild-type (white bars) and the *nagZ* mutant (grey bars). CFU counts were determined and normalized to the untreated control for each condition. Treatment was performed in PBS for 1 h. Data represent means ± SE (n = 3).


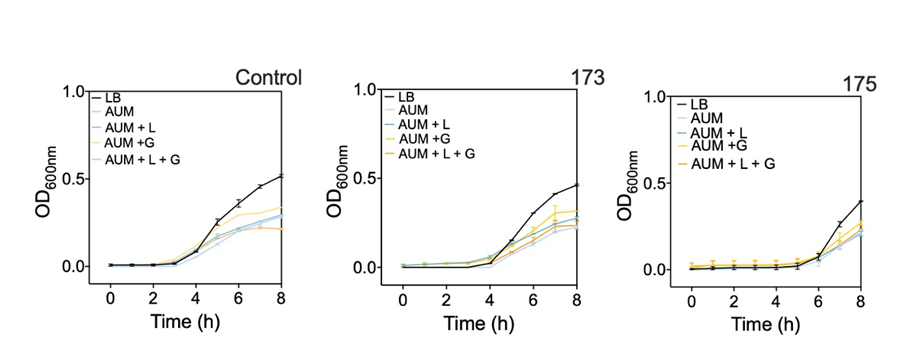


**Figure S29. Growth curve of treated *E. coli* persister cells during resuscitation in different media.** Resuscitation of AMP UPEC persisters in LB (black), AUM (light blue), AUM + L (AUM with 0.04% L- alanine, teal), AUM + G (AUM with 0.4% glucose, yellow), and AUM + L + G (AUM with 0.04% L- alanine and 0.4% glucose, orange). Glucose and L-alanine are known persister resuscitation factors ^1^.The growth of each sample was followed by monitoring OD_600_ over 32 h.


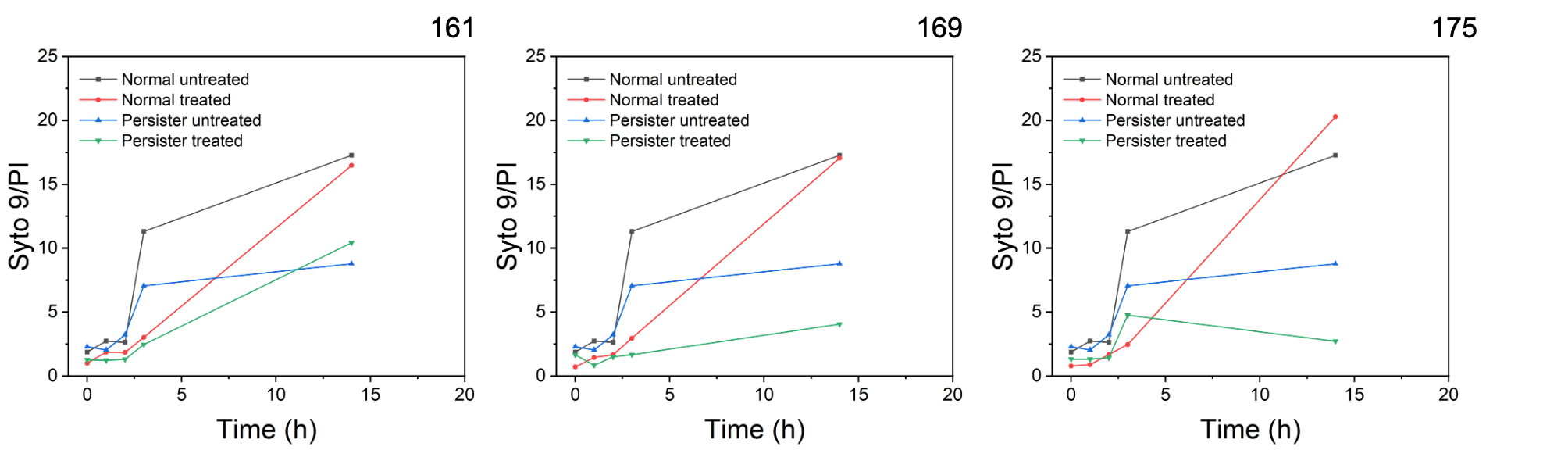


**Figure S30. Syto 9/PI signal ratio of *E. coli* HM22 cells after treatment with compounds 161, 169 and 175.** Syto 9 and PI signals were measured with flow cytometry.

**References**

1 Yamasaki, R., Song, S., Benedik, M. J. & Wood, T. K. Persister Cells Resuscitate Using Membrane Sensors that Activate Chemotaxis, Lower cAMP Levels, and Revive Ribosomes. *iScience* **23**, 100792 (2020). https://doi.org/10.1016/j.isci.2019.100792
